# Model Construction and Analysis of Respiration in *Halobacterium salinarum*

**DOI:** 10.1371/journal.pone.0151839

**Published:** 2016-03-24

**Authors:** Cherryl O. Talaue, Ricardo C. H. del Rosario, Friedhelm Pfeiffer, Eduardo R. Mendoza, Dieter Oesterhelt

**Affiliations:** 1 Institute of Mathematics, University of the Philippines, Diliman, Quezon City, Philippines; 2 Max Planck Institute of Biochemistry, Department of Membrane Biochemistry, Martinsried, Germany; University of Bern, SWITZERLAND

## Abstract

The archaeon *Halobacterium salinarum* can produce energy using three different processes, namely photosynthesis, oxidative phosphorylation and fermentation of arginine, and is thus a model organism in bioenergetics. Compared to its bacteriorhodopsin-driven photosynthesis, less attention has been devoted to modeling its respiratory pathway. We created a system of ordinary differential equations that models its oxidative phosphorylation. The model consists of the electron transport chain, the ATP synthase, the potassium uniport and the sodium-proton antiport. By fitting the model parameters to experimental data, we show that the model can explain data on proton motive force generation, ATP production, and the charge balancing of ions between the sodium-proton antiporter and the potassium uniport. We performed sensitivity analysis of the model parameters to determine how the model will respond to perturbations in parameter values. The model and the parameters we derived provide a resource that can be used for analytical studies of the bioenergetics of *H. salinarum*.

## Introduction

The archaeon *Halobacterium salinarum* thrives in extremely salty environments (around 4M) using three bioenergetic processes: photosynthesis, respiration and fermentation of arginine. When sufficient light is available, it uses bacteriorhodopsin, a membrane-bound retinal protein which drives the only known non-chlorophyll photosynthetic system, to enhance the membrane potential (ΔΨ) [1–6]. In the absence of light, the respiratory pathway is used by the organism to enhance the membrane potential. It can also use the arginine pathway as an energy source [2, 7, 8]. Photosynthesis and respiration produce energy by enhancing the membrane potential which then drives phosphorylation, while fermentation of arginine produces energy by substrate level phosphorylation.

Compared to its photosynthetic pathway, much less attention has been devoted to its respiratory pathway. We therefore constructed a mathematical model of its respiration to help address this lag in knowledge. In particular, we endeavored to show that the known components of the respiratory pathway and the experimental data on bioenergetics taken within the last 30 years are consistent. We achieved this by showing that the diverse set of experimental data could be modeled by a single system of ordinary differential equations (ODEs). Some of the data are from photo-phosphorylation experiments, such as the maximum value of the proton motive force, the rate of membrane potential generation, and maximum internal ATP concentration. Our hypothesis that data on light-driven proton transport by bacteriorhodopsin can be used for modeling the respiratory pathway is in accordance with Mitchell’s chemiosmotic theory that the coupling of ATP synthesis and the ion pumps is via the proton motive force.

**Fig 1:**
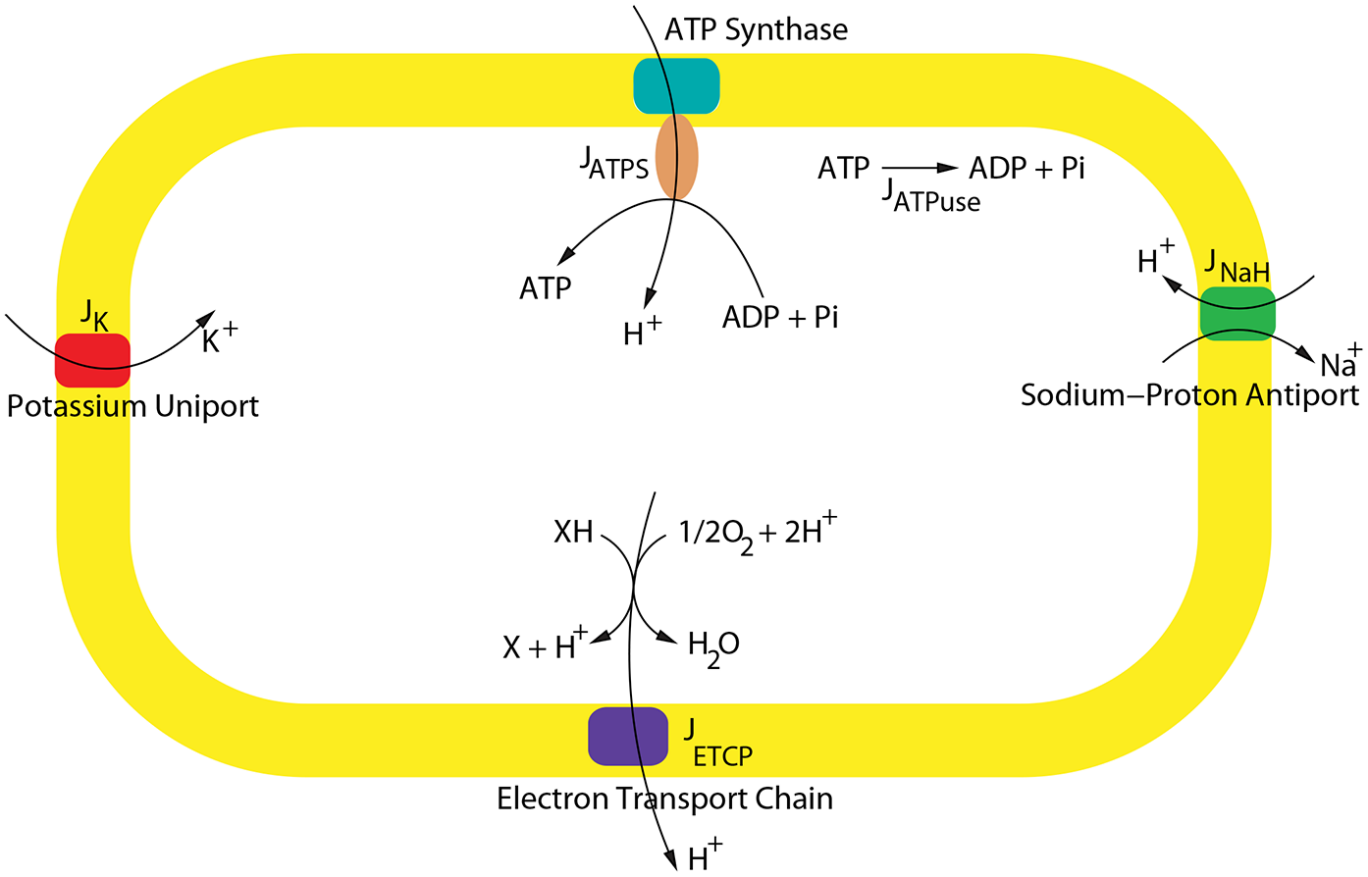
Bioenergetic components of the respiratory model. The figure depicts the fluxes and variables of the oxidative phosphorylation model. The flux of the electron transport chain energizes the membrane which is used by the ATP synthase to create ATP. The potassium uniport creates a potassium gradient that can be used by the cell as a battery in darkness [3], while the sodium-proton antiport is used to regulate internal pH. X: unknown electron donor.

The model we present consists of the following bioenergetic components: the ATP synthase, the sodium-proton antiport, the potassium uniport and the electron transport chain (ETC) (Fig 1). In Fig 1, we adopted the notation in [9] where the symbol X was used to denote the unknown electron donor in the electron transport chain. This unknown electron donor is one of the questions still to be answered regarding the respiration in this organism. Experiments in *H. salinarum* have indicated that NADH is not oxidized by a type I dehydrogenase but by a non-homologous type II NADH dehydrogenase incapable of proton translocation [10]. However, the conservation of eleven complex I subunits in the genome of *H. salinarum* with high levels of sequence similarity indicates that the complex is functional [9, 11]. This complex I analog lacks a NADH-specific acceptor module and thus it is possible that it actually accepts electrons from another donor molecule [9]. Hence, as also done in [9], we denoted the unknown electron donor as XH which is oxidized into X.

Although a number of mathematical models for mitochondrial respiration (see for example [12–16]) or respiration in prokaryotes [17] are available, our model is the first ODE-based model of respiration in *H. salinarum* whose parameters were fitted to experimental data. We have previously shown using Petri nets that the bioenergetic components of our model (Fig 1) constitute a basic set of processes in the respiratory pathway of *H. salinarum* [18]. The Petri net model in [18] was discrete and time-independent. The ODE model, on the other hand, can simultaneously capture the dynamic changes of the different components of the respiratory pathway, and hence can be used as a tool for testing hypotheses regarding how each bioenergetic component can impact the dynamics of the whole system. The model can also be used as a basic model upon which more complicated models can be built by adding other bioenergetic components.

The sodium-proton antiport is an important component of the bioenergetic system since it regulates the internal pH and salt concentrations. Furthermore, it has been shown that the potassium ions that enter the cell via the potassium uniport are charged balanced by the release of sodium ions [3]. Thus, the antiport plays a role in building the potassium gradient which can be used as a battery in darkness to charge the membrane [3]. Although it has been previously claimed that the sodium-proton antiport in *H. salinarum* is electrogenic [19, 20], to date the stoichiometry of the antiport has not been determined, either from biochemical studies or from genome sequence analysis. Therefore, we constructed two models, one with an electrogenic antiport and another with an electroneutral antiport. By performing simulations of each model, we were able to study the impact of electrogenicity of the antiport on the bioenergetics of *H. salinarum*.

We also present a resource for bioenergetics modeling in *H. salinarum* by summarizing quantitative cellular measurements and calculating the basic building blocks of the cell. These values are necessary in quantitative modeling to convert different bioenergetics measurements such as nmol ATP/mg protein to mmol ATP/kg water.

## Results and Discussion

### Electrogenic and electroneutral models of respiration in *H. salinarum*

The mathematical model was based on the bioenergetic processes involved in oxidative phosphorylation of *H. salinarum* (see Fig 1). The electron transport chain involves a series of reactions whose net effect is to pump protons outside the membrane, resulting in the enhancement of the proton motive force (pmf). The reaction flux of the ETC was denoted by *J*_*ETCP*_ in Fig 1. Another component is the ATP synthase which uses the pmf to translocate protons from the outer to the inner side the membrane, and in the process drives ATP-synthesis (Fig 1, *J*_*ATPS*_). We also included the potassium uniport in the model, which uses the pmf to drive potassium ions inside the cell (Fig 1, *J*_*K*_). The importance of the potassium uniport in bioenergetics was discovered in [3] where it was shown that the potassium gradient created by the uniport can be used as a battery to enhance the membrane potential. The sodium-proton antiport (Fig 1, *J*_*NaH*_) regulates the internal pH and is one mechanism used by the microbe to survive in extremely salty environment [21]. The final process that we considered in the model was the consumption of ATP via non-vectorial substrate hydrolysis, i.e., not via the reversal of the ATP synthase (Fig 1, *J*_*ATPuse*_). Only oxidative phosphorylation of the organism under darkness was considered and hence light-driven processes such as the ion pumping of bacteriorhodopsin and halorhodopsin were not included. Furthermore, ionophores and ion leaks were not explicitly modeled. The pathway in Fig 1 contains the components we deemed necessary to achieve our goal of explaining data on oxygen consumption, dynamics of ATP synthesis and the enhancement of the proton motive force.

The model consists of five differential equations that model the rate of change of the concentrations of five *dependent variables*: $\left[{\text{H}}_{\text{i}}^{+}\right]$, $\left[{\text{K}}_{\text{i}}^{+}\right]$, $\left[{\text{Na}}_{\text{i}}^{+}\right]$, [ATP] and ΔΨ (see Table 1 for a description of the variables). The model also includes variables that remain constant throughout the simulation, denoted as *independent variables*, and can be used as input to change properties of the system (Table 1). Finally, the model includes *algebraic variables* which are expressed in terms of the dependent and independent variables and which have physical interpretation (Table 1, Methods).

**Table 1:** The model variables.

Variable	Description	Units	Equation number or Constant Value
Dependent Variables			
[${\text{H}}_{\text{i}}^{+}$]	intracellular proton concentration	mol/liter	Eq (1)
[ATP]	intracellular ATP concentration	mol/liter	Eq (1)
[${\text{K}}_{\text{i}}^{+}$]	intracellular potassium concentration	mol/liter	Eq (1)
[${\text{Na}}_{\text{i}}^{+}$]	intracellular sodium concentration	mol/liter	Eq (1)
ΔΨ	membrane potential	volts	Eqs (1) and (2)
Independent Variables			
[P_i_]	intracellular inorganic phosphate concentration	mol/liter	0.035 mol/liter
[O_2_]	intracellular oxygen concentration	mol/liter	0.1080 mol/liter
[A_tot_]	fixed sum of [ATP] and [ADP]	mol/liter	0.0023 mol/liter
[XH]	electron donors	mol/liter	5.8824 × 10^−3^ mol/liter
[ATPS]	ATP synthase concentration	mol/liter	4.9816 × 10^−5^ mol/liter
[ETCP]	combined concentration of complexes III and IV	mol/liter	6.6422 × 10^−5^ mol/liter
Algebraic Variables			
[${\text{H}}_{\text{o}}^{+}$]	extracellular proton concentration	mol/liter	Eq (3)
[${\text{K}}_{\text{o}}^{+}$]	extracellular potassium concentration	mol/liter	Eq (4)
[${\text{Na}}_{\text{o}}^{+}$]	extracellular sodium concentration	mol/liter	Eq (5)
pmf	proton motive force	volts	Eq (6)
Δ*μ*_H^+^_	free energy to transfer a mole of protons	joules/mol	Eq (8)
Δ*μ*_K^+^_	free energy to transfer a mole of potassium	joules/mol	Eq (11)
Δ*μ*_Na^+^_	free energy to transfer a mole of sodium	joules/mol	Eq (10)
ΔpH	pH gradient		Eq (7)
ΔpNa	Na^+^ gradient		Eq (9)
ΔpK	K^+^ gradient		Eq (12)
[ADP]	cellular ADP concentration	mol/liter	[A_tot_]- [ATP]

The units of the model variables, and the equations which define them, are given in the last two columns. The dependent variables are modeled by differential equations, the independent variables have fixed values, and the algebraic variables are expressed in terms of the dependent and independent variables. The derivation of the constant values of the independent variables are given in S1 File.

To create the system of differential equations, the fluxes of the reactions in Fig 1 were expressed in terms of the variables, and then the rate of change of each dependent variable was modeled as the net sum of the reactions which affect that variable. Each flux associated with a variable either increases or decreases the rate of that variable. The details of how we modeled each flux are given in Methods. Since one application of our model is to perform an analysis of the electrogenicity of the sodium-proton antiport in *H. salinarum*, we created two models, one where the sodium-proton antiporter exchanges one sodium ion with one proton, and the other includes the ratio of proton to sodium ions as a parameter to be estimated. The electroneutral (EN) model is given by the following system of differential equations

$$\begin{array}{ccc} \frac{d[H_{i}^{+}]}{dt} & = & n_{\text{syn}}J_{ATPS}+J_{NaH}^{n}-J_{ETCP}\\ \frac{d[\text{ATP}]}{dt} & = & J_{ATPS}-J_{ATPuse}\\ \frac{d[K_{i}^{+}]}{dt} & = & J_{K}\\ \frac{d[{\text{Na}}_{i}^{+}]}{dt} & = & -J_{NaH}^{n}\\ \frac{d\Delta \Psi }{dt} & = & \beta_{mempot}(J_{ETCP}-n_{\text{syn}}J_{ATPS}-J_{K})\;. \end{array}$$ (1)

The fixed parameter *n*_syn_ denotes the number of protons involved in the phosphorylation of one molecule of ADP by the ATP synthase, and *β*_*mempot*_ is a parameter to be estimated (see Methods). For the model with an electrogenic sodium-proton antiporter (EG), the differential equations for [ATP], $\left[{\text{K}}_{\text{i}}^{+}\right]$ and $\left[{\text{Na}}_{\text{i}}^{+}\right]$ are of the same form as in Eq (1), except that the flux expression for the sodium-proton antiporter is different since it is driven by both the pH gradient and the membrane potential (Methods). Furthermore, the rate of change of the protons ($d[{\text{H}}_{\text{i}}^{+}]/dt$) and membrane potential (*d*ΔΨ/*dt*) are now affected by the flux from the antiport, and the equations are given below

$$\begin{array}{ccc} \frac{d[H_{i}^{+}]}{dt} & = & n_{\text{syn}}J_{ATPS}+n_{NaH}J_{NaH}^{e}-J_{ETCP}\\ \frac{d\Delta \Psi }{dt} & = & \beta_{mempot}(J_{ETCP}-n_{\text{syn}}J_{ATPS}-J_{K}-\left(n_{NaH}-1\right)J_{NaH}^{e})\;. \end{array}$$ (2)

The parameter *n*_*NaH*_ denotes the electrogenic antiporter ratio of protons to sodium ions. In Eqs (1) and (2), we used the superscripts *n* and *e* to identify the fluxes that differ between the two models (*n* for electroneutral, *e* for electrogenic). The models include unknown parameters (8 for the electroneutral model and 9 for electrogenic model) which were estimated from data (see Methods).

### Parameter estimation yields models that explain data

We estimated the values of the model parameter Eq (1) by minimizing a cost function that measures the difference between model output and experimental data (Methods; Eq (25)). All experimental data were taken from the literature (see Methods). To minimize Eq (25), we used two optimization algorithms: the Nelder Mead algorithm provided in Matlab (fminsearch) and a Matlab implementation of simulated annealing (J. Vandekerckhove, Matlab Central). These algorithms required an initial guess for the parameter values for which we supplied two sets: for the first set, all parameter values were taken to be one and for the second set, we performed intensive manual search to obtain a set of parameters that provided a correct qualitative behavior of the model (Methods). Note also that initial conditions of the 5 dependent variables have to be provided to the numerical ODE solver that is called by the optimization algorithms and our derivation of the initial conditions are given in the Methods section.

#### Electroneutral Model

**Fig 2:**
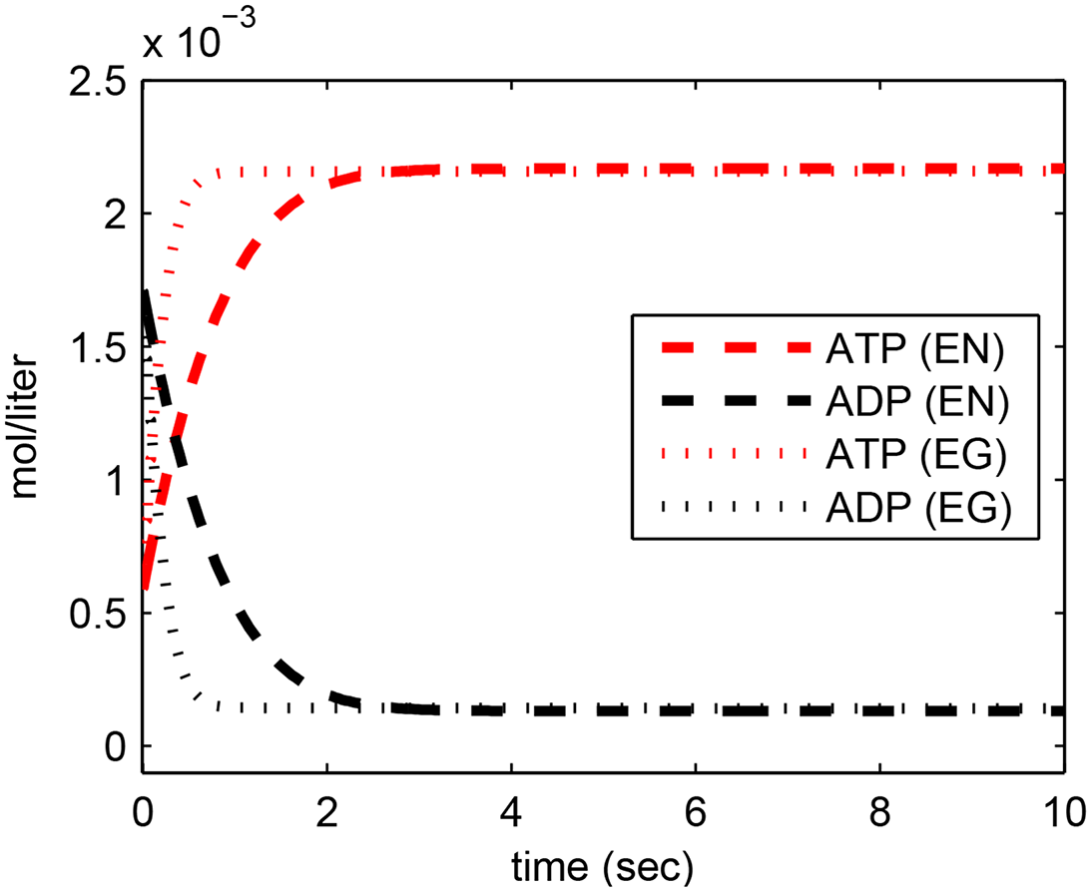
Model output for intracellular ATP and ADP concentrations. Lines labeled EN: electroneutral model using set EN5 in Table 2. Lines labeled EG: electrogenic model using parameter set EG1 in Table 3.

The best parameter set we obtained for the electroneutral model (set EN5 in Table 2) predicted that ATP reaches the maximum intracellular concentration of around 2.2 mmol/liter (Fig 2, lines labeled (EN)). This maximum ATP value was converted to 7.5 nmol/mg protein using our scripts for unit conversion (for details see Methods, S1 File and Matlab scripts for conversion provided in S1 Matlab Code). Our maximum ATP value was consistent with previous measurements [1], and was attained in 3.6 seconds. Hence the net rate of ATP production is 2.1 nmol ATP/second/mg protein (note that this is not the rate of ATP synthesis but net ATP production rate). This rate is about 5.7 times faster than the experimentally measured maximum rate of phosphorylation under illumination (0.37 nmol ATP/second/mg protein; see Fig 2 in [1]). One possible source of the difference is that in [1], the rate was measured at a high light intensity of 25 mW/cm^2^ without oxygen, and hence the ATP synthase was rate limiting (i.e., it is at its maximum catalytic activity) and ATP synthase regulation could play a role in the experiments, which was not modeled in this study. Another possible reason is that our model did not take into account enzyme saturation kinetics.

**Table 2:** Parameter estimation results for the electroneutral model.

	EN1	EN2	EN3	EN4	EN5
*β*_*ATPS*_	1.310	0.020	0.017	0.020	0.042
*γ*_*ATPS*,*ADP*_	0.525	0.090	0.123	0.375	0.174
*β*_*ETCP*_	0.732	0.046	0.048	0.101	0.085
*γ*_*ETCP*,*pmf*_	0.679	0.180	0.175	0.235	0.355
*α*_*NaH*_	1.562	0.800	0.240	0.692	3.693 × 10^−4^
*α*_*K*_	0.971	1.500 × 10^−8^	1.400 × 10^−8^	0.203	0.601
*α*_*ATPuse*_	0.911	0.600	0.771	0.888	0.779
*β*_*mempot*_	0.870	1.000 × 10^2^	1.055 × 10^2^	1.002 × 10^2^	1.056 × 10^2^
Cost, Eq (25)	0.314	0.631	0.041	0.255	0.193

**Fig 3:**
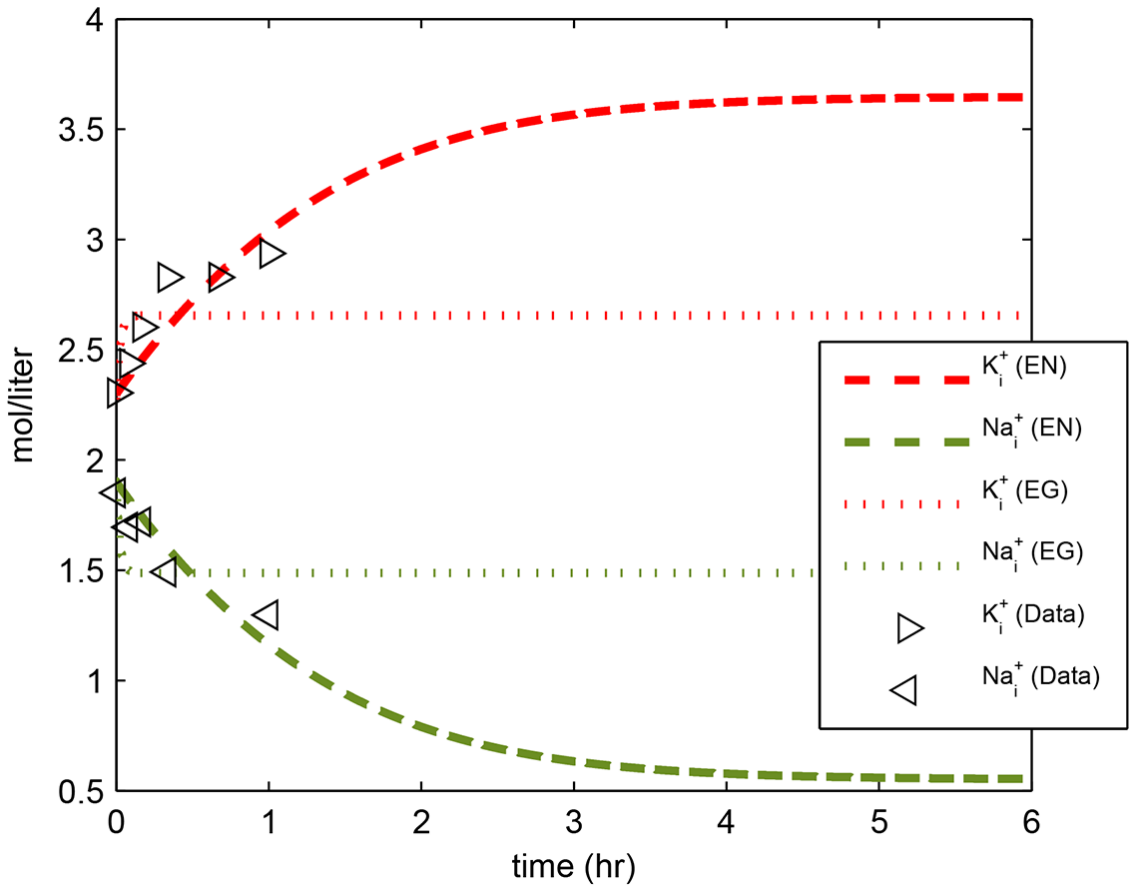
Model output for intracellular potassium and sodium ions. Triangles: experimental data from [3]. Lines labeled EN: electroneutral model using set EN5 in Table 2. Lines labeled EG: electrogenic model using parameter set EG1 in Table 3.

**Fig 4:**
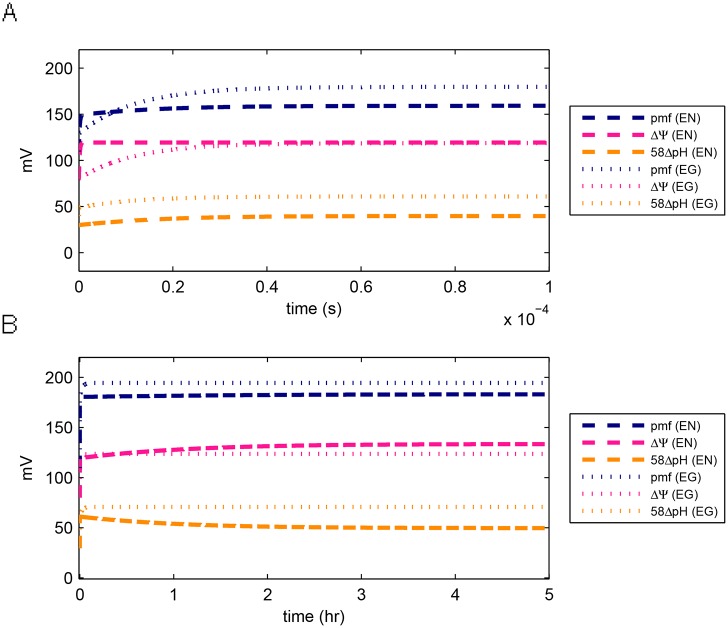
Model output for the proton motive force (pmf), ΔΨ and ΔpH. The dynamics of the variables are plotted for a short interval (A) and a long interval (B). Lines labeled EN: electroneutral model using set EN5 in Table 2. Lines labeled EG: electrogenic model using parameter set EG1 in Table 3. 58ΔpH: ΔpH converted to mV for comparison with ΔΨ and pmf. At the last time point in (A), the electroneutral model has reached the following percentage of the steady state values: 87% of the pmf, 89% of ΔΨ and 80% of 58ΔpH, while the electrogenic model has reached 92% of the pmf, 96% of ΔΨ and 86% of 58ΔpH. The short interval (A) was plotted in logarithmic scale in Fig A in S1 File to show when the jumps in values occurred.

Set EN1: parameter values obtained using simulated annealing with a vector of ones provided as initial guess. Set EN2: manually obtained (via trial and error) parameter values to be used as initial guess. Sets EN3 and EN4: refinement of the manually obtained parameters using two optimization algorithms, Nelder Mead and Simulated Annealing, respectively. Set EN5: the best set of parameter values for the electroneutral model was obtained by performing another round of optimization on set EN3 using Simulated Annealing. Set EN5 was used for the model output in Figs 2 to 4.

The model output for potassium uptake and sodium release during one hour followed the measurements in [3] (Fig 3, lines labeled (EN)).

The dynamics of the membrane potential, 58ΔpH (this is ΔpH converted to mV for comparison with pmf and ΔΨ) and their sum (pmf) were plotted for a short time interval of 1 × 10^−4^ seconds (Fig 4A) and for a longer time interval of 5 hours at which the values were almost at the steady state (Fig 4B). In photo-phosphorylation studies, bacteriorhodopsin was found to energize the membrane to its maximum potential within milliseconds [4, 22]. This rate has not been experimentally measured in oxidative phosphorylation. The proton motive force, ΔΨ and 58ΔpH of the electroneutral model showed a rapid increase in values (initial time points in Fig 4A) followed by a slow increase in values. After reaching the maximum value for 58ΔpH (at around 5.4 seconds in Fig 4B), the membrane potential continued a slow increase while 58ΔpH showed a slow decrease. We re-plotted Fig 4 in logarithmic scale and found that ΔΨ had a jump at *t* = 6 × 10^−7^ and 58ΔpH had a jump at *t* = 6 × 10^−5^ (Fig A in S1 File). At the last time point in Fig 4A, these rapid jumps have contributed to 87% of the pmf. These dynamics were very fast compared to the measured time it takes bacteriorhodopsin to energize the pmf (milliseconds). When the measurement of the time it takes the pmf to be maximally energized by the respiratory pathway becomes available, then it would be possible to re-estimate model parameters that would fit this correct time for the rapid jump in pmf. Since this is currently not a goal in our study, we decided not to search for parameters that would take a longer time to reach the jump in the values of ΔΨ and 58ΔpH.

The maximum pmf of the model using parameter set EN5 was less than 200 mV (Fig 4, lines labeled (EN)), which was lower than the experimentally measured maximum value of 280 mV attained via bacteriorhodopsin [4]. To determine if the model could achieve a higher pmf, we performed additional numerical estimation computations and found parameter sets EN2, EN3 and EN4 (Table 2) that yielded a maximum pmf near 280 mV (Figs C, D and E in S1 File). However the better fit to the maximum pmf (compared to parameter set EN5) resulted in a poorer fit to ${\text{Na}}_{\text{i}}^{+}$ and ${\text{K}}_{\text{i}}^{+}$ (Figs C, D and E in S1 File).

Our model exhibited the following observed behavior of bacteriorhodopsin-driven pmf dynamics observed in Ref [4, Fig 2]): (i) after the maximum membrane potential has been reached, the sum of the membrane potential and ΔpH remains constant, and (ii) although their sum is constant, the membrane potential and 58ΔpH continues to vary (Fig 4B). However, the concavity of our model was opposite to the observation in [4]—the model’s membrane potential was concave down while 58ΔpH was concave up. Note that the measurements in [4] were done without oxygen over a 15 minute period. It is possible that there is a difference of the time dependence of the membrane potential used by proton translocating processes and other ion translocating processes between respiration without light and light-driven phosphorylation without oxygen. Such a difference could cause the opposite concavity of our model. Another possible simpler explanation of the opposite concavity is that some bioenergetic components were not included in our model.

A more pronounced decrease in membrane potential and increase in ΔpH was exhibited by our model (parameter sets EN2 and EN3; Figs C and D in S1 File), although the concavity was still opposite experimental measurements.

#### Electrogenic Model

For the respiratory system with an electrogenic antiporter, we performed the same parameter estimation strategy as in the electroneutral model (Methods). We chose parameter set EG1 from Table 3 to plot the model output (Figs 2 to 4). The electrogenic model achieved the same steady state ATP and ADP concentrations as the electroneutral model, but with a higher rate of production (Fig 2). It achieved a higher maximal value of pmf, with dynamics comparable to the electroneutral model (Fig A in S1 File), but it did not exhibit the decrease in membrane potential and increase in 58ΔpH (Fig 4). Parameter sets EG2 and EG3 show that the electrogenic model is potentially capable of exhibiting the experimentally observed phenomenon that the sum of the membrane potential and 58ΔpH remains constant after a transient interval (Figs H and I in S1 File; and note that the value of pmf has a discontinuity or jump in Fig H around *t* = 1 hr). These results only give confidence to the capability of the model to exhibit this phenomenon, but the parameter values themselves are not physically meaningful since although the pmf remained within 280 mV, the non-steady state values of 58ΔpH reached negative voltages, while the membrane potential increased beyond experimentally measured values.

**Table 3:** Parameter estimation results for the electrogenic model.

	EG1	EG2	EG3	EG4	EG5
*β*_*ATPS*_	0.835	0.020	0.018	0.027	0.106
*γ*_*ATPS*,*ADP*_	0.477	0.090	0.108	0.204	0.207
*β*_*ETCP*_	1.089	0.046	0.054	0.019	0.018
*γ*_*ETCP*,*pmf*_	0.635	0.180	0.180	0.154	0.224
*α*_*NaH*_	1.088	0.800	0.988	0.997	1.055
*α*_*K*_	1.152	1.500 × 10^−8^	7.353 × 10^−9^	0.117	0.139
*α*_*ATPuse*_	1.019	0.600	0.868	0.493	0.809
*β*_*mempot*_	0.754	1.000 × 10^2^	1.022 × 10^2^	9.944 × 10^1^	1.025 × 10^2^
*n*_NaH_	0.76	2.0	1.54	1.90	1.45
Cost, Eq (25)	0.184	0.720	0.023	0.295	0.320

Sets EG1-EG5 were obtained using the same parameter estimation strategy as for sets EN1-EN5, respectively, of the electroneutral model. Set EG1 was used for the model output in Figs 2 to 4.

The sodium and potassium ions of the electrogenic model showed different dynamics than the electroneutral model and achieved a lower level of ${\text{K}}_{\text{i}}^{+}$ and a higher level of ${\text{Na}}_{\text{i}}^{+}$ (Fig 3). We were able to find a set of parameters that could fit ${\text{Na}}_{\text{i}}^{+}$ and ${\text{K}}_{\text{i}}^{+}$ well (parameter set EG5, Fig K in S1 File), but the parameter values were not physically meaningful since the model yielded negative 58ΔpH values. As in our conclusion above, this validates the capability of the model to fit the sodium and potassium data, although we will not use these parameters in other analyses as they are not physically meaningful.

Parameter set EG1, which was the only set we found that could model the data, had a proton to sodium ratio of 0.76 (Table 3). This is opposite to the expected ratio of greater than one. We performed more numerical calculations to obtain an electrogenic model with ratio >1 that could fit the data, including other optimization algorithms (genetic algorithms and Newton-type gradient based algorithms), however, all failed to converge (data not shown). We believe that instead of implying that an electrogenic antiport has a ratio less than one, this result has implications about modeling an electrogenic system: an electrogenic system is more sensitive than an electroneutral system to processes that affect the pmf. This is supported by our result below in parameter sensitivity analysis which showed that the electrogenic model is 5 orders of magnitude more sensitive to the proton to sodium ratio than the other model parameters (see parameter sensitivity result below).

### Estimation of consumed oxygen

**Fig 5:**
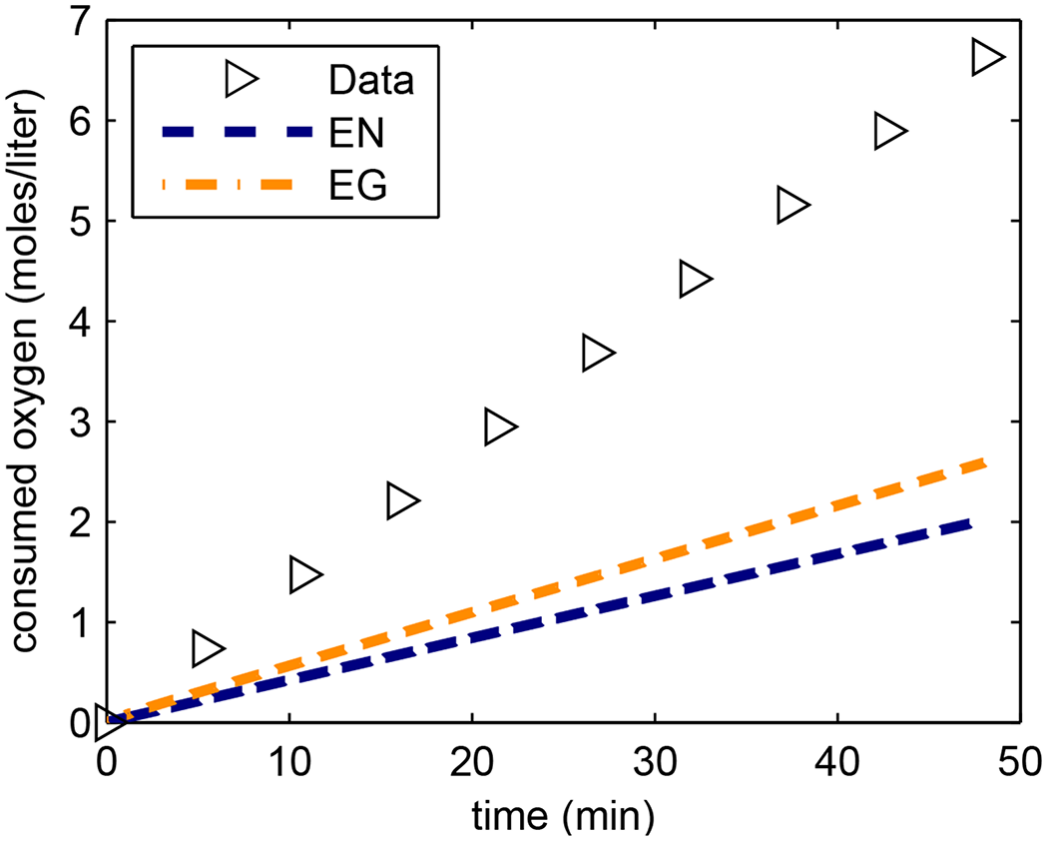
Model output for oxygen consumption. Oxygen consumption of the electroneutral model using parameter set EN5 and electrogenic model using parameter set EG1. Data taken from [24].

To estimate the amount of consumed oxygen, we used the flux of protons through the electron transport chain given by *J*_*ETCP*_. Although the individual stoichiometry of the proton translocating processes in the respiratory chain of *H. salinarum* is still unknown, data from bulk measurements indicate an approximate value of around 10 protons per O_2_ [23]. We thus used this ratio and computed the amount of consumed oxygen by integrating the flux *J*_*ETCP*_ with respect to time. Oxygen consumption in *H. salinarum* has been measured in [24], where it was found that oxygen consumption was constant, both in the absence or presence of light (but with lower consumption rate in light). We compared our model output with the consumed oxygen data (see S1 File for details on how we converted the experimental data to agree with the model units), and plotted the results in Fig 5. Due to the rapid stabilization of the pmf, *J*_*ETCP*_ rapidly decreased its value, hence its integral was linear. The electroneutral and electrogenic models showed slower oxygen consumption than experimental data, but the values were still within physiological ranges. A possible explanation that could contribute to the lower oxygen consumption of the model was that oxygen was only used for ATP production and we did not take into account uncoupled respiration (independent of pmf).

### Cellular parameters of *H. salinarum*

**Fig 6:**
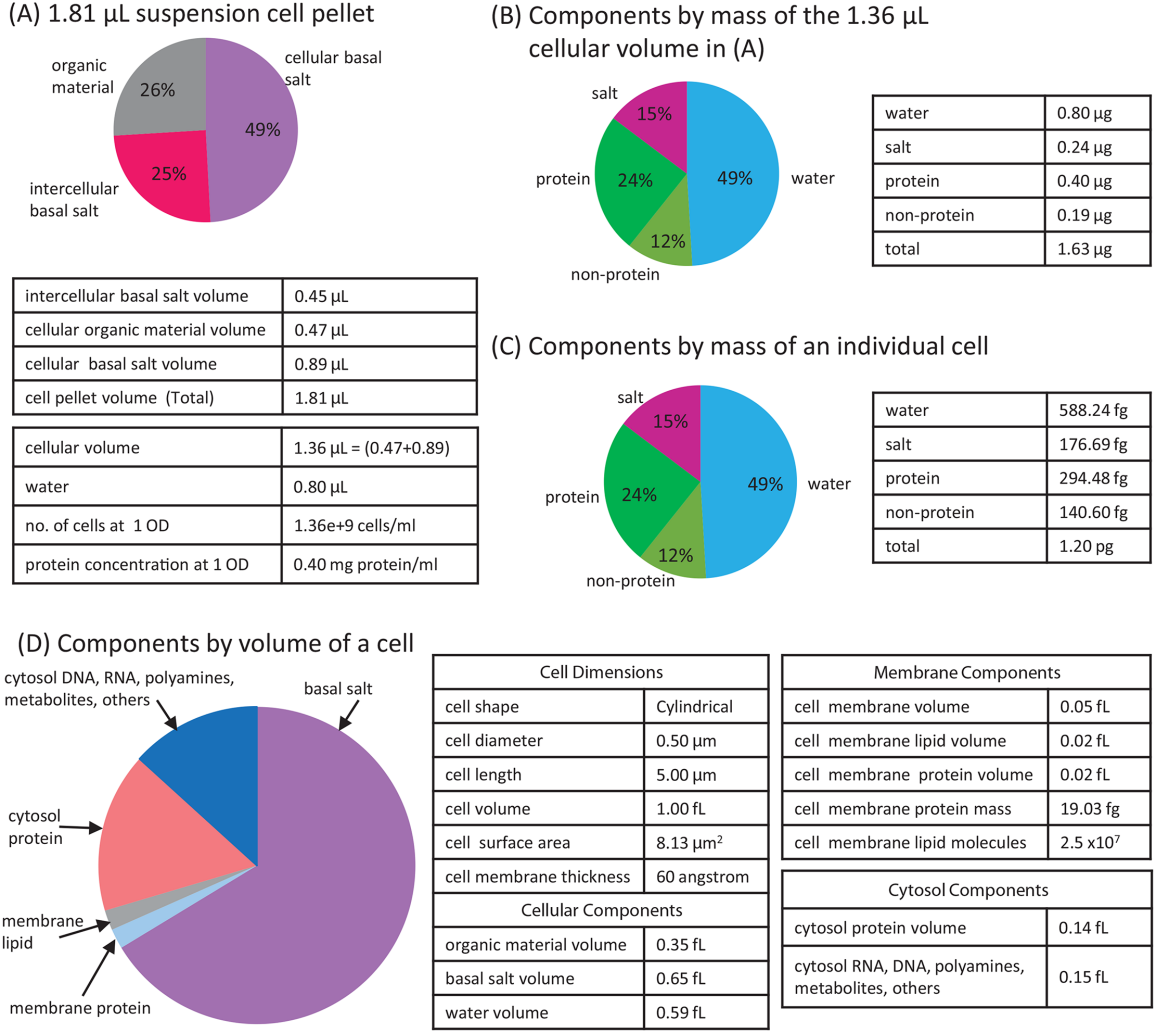
The building blocks of a cell obtained by harmonizing the different data gathered from literature [25, 26]. A 1 ml OD cell suspension contains 1.81 *μ*L cell pellet and 1.36 × 10^9^ halobacterial cells. (A) The 1.81 *μ*L cell pellet consists of 0.47 *μ*L cellular organic material, 0.45 *μ*L cellular basal salt and 0.89 *μ*L inter-cellular basal salt. The total cell volume (1.36 *μ*L from the sum of organic material and cellular basal salt) contains 0.80 *μ*L water. (B) Using a buoyant density of 1.2 mg/mL for the cells [25, 26], the 1.36 *μ*L cell volume is 1.63 *μ*g. This consists of 0.80 *μ*g water and other components. (C) Assuming that a cell pellet contains 1.36 × 10^9^ halobacterial cells, then an individual cell has a mass of 1.2 pg. (D) The components by volume of a cell is given. See S1 File for more details. Units: *μ*L = micro liters, pg = pico grams, fL = femto liters.

During the process of setting up the mathematical model, we had to gather data on the cellular parameters of *H. salinarum*, e.g., number of cells in a 1 mL suspension, individual cell volume, volume of water, salt and organic material in a cell. Given a 1 mL cell suspension at 1 OD, we computed the components of the suspension (salt, water, protein, no. of cells, etc.) both by volume and by mass (Fig 6A and 6B). For each individual cell, we computed the components by mass and by volume (Fig 6C and 6D). The details of how we derived these values are presented in S1 File. These values can be used as a resource when working on quantitative models of *H. salinarum*. In our case, we used these cellular and medium properties to convert experimental data from different cellular concentration units, since in *H. salinarum* bioenergetics experiments, the concentrations of the substances had been reported using different units (e.g., mmol substance/kg water, or nmol/liter, or nmol/mg protein). We provide a set of Matlab scripts that can be used to perform conversion of different concentration units (S1 Matlab Code).

### Parameter Sensitivity Analysis and Parameter Ranking

Parameter sensitivity analysis of the model, which quantifies the response of the model output to minute perturbations in the model parameters and initial conditions, can be performed using steady state or time-dependent methods. In this study, we performed time-dependent parameter sensitivity analysis where we computed the response of the system to infinitesimal perturbations in parameter values at each time instance [27]. In order to rank the parameters according to their sensitivity, we computed the Fisher Information Matrix (FIM) (see Methods).

The electrogenic model shows a very high sensitivity to the ratio of the proton to sodium ions. The ratio parameter *n*_*NaH*_ of the electrogenic model has an FIM value of 5.48 × 10^20^, while the other 8 parameters, for both the electroneutral and electrogenic models, have FIM values that range between 8.19 × 10^9^ to 3.65 × 10^15^ (Table 4). These other 8 parameters are common between the electroneutral and electrogenic models, and for each parameter, the difference between the two models is less than 2 orders of magnitue (Table 4). In contrast, the electrogenic model is at least 5 orders of magnitude more sensitive to *n*_*NaH*_ than the 8 common parameters. This result supports our observation above that the electrogenic model is sensitive to processes that affect pmf generation, which is probably one reason that explain the difficulties we encountered in fitting the electrogenic model to data. We calculated the parameter sensitivities of the models using the other parameter sets and an even higher sensitivity of the electrogenic model to parameter *n*_*NaH*_ was found (Tables A and B in S1 File).

**Table 4:** Parameter sensitivities.

Rank	Electroneutral		Electrogenic	
1	*γ*_*ETCP*,*pmf*_	5.95 × 10^15^	*n*_*NaH*_	5.48 × 10^20^
2	*γ*_*ATPS*,*ADP*_	3.65 × 10^15^	*γ*_*ETCP*,*pmf*_	1.99 × 10^15^
3	*β*_*ETCP*_	2.36 × 10^15^	*γ*_*ATPS*,*ADP*_	1.81 × 10^15^
4	*β*_*ATPS*_	2.34 × 10^15^	*β*_*ATPS*_	4.22 × 10^14^
5	*α*_*ATPuse*_	6.47 × 10^13^	*β*_*ETCP*_	4.14 × 10^14^
6	*α*_*K*_	2.03 × 10^13^	*α*_*ATPuse*_	8.46 × 10^13^
7	*α*_*NaH*_	2.75 × 10^12^	*α*_*NaH*_	7.01 × 10^11^
8	*β*_*mempot*_	8.19 × 10^9^	*α*_*K*_	4.48 × 10^10^
9	−	−	*β*_*mempot*_	1.77 × 10^10^

The parameter sensitivities (FIM) obtained from Eq (27). The nominal parameter values were Set EN5 for the electroneutral model and Set EG1 for the electrogenic model.

## Conclusions

We have shown that the respiratory pathway of *H. salinarum* can be modeled using a simple set of differential equations that fit experimental data from various experimental conditions. This confirms our hypothesis that the knowledge on the bioenergetics of this organism gained within the last 30 years is consistent. The model we presented can now be combined with other bioenergetic processes such as light-driven phosphorylation or fermentation of arginine. In particular, it can be combined with the model we have previously presented on membrane potential generation of bacteriorhodopsin [28]. An interesting outcome of such a combined model is to study the inhibition of respiration by photo-phosphorylation and to compare the results with data from [24].

A detailed network of the respiratory pathway of *H. salinarum* has been proposed in [9]. However, their network did not include the sodium-proton antiport and the potassium uniport and moreover, they did not consider an ODE model of the respiratory pathway. Another detailed respiratory pathway was presented in [17], but only steady state analysis was performed, the model did not include the sodium-proton antiport and potassium uniport, and the steady state parameters were optimized for purple non-sulfur bacteria. In our model, we did not incorporate all component details of the pathways in [9] and [17] as these would lead to an intractable parameter estimation problem. As one extension of our study is to embed the model within larger bioenergetic systems, then the use of only the main respiratory components is important as its incorporation within a larger model would necessitate a new round of parameter estimation calculations. We have previously performed a discrete Petri net analysis (where the variables have discrete values and the system does not evolve in time) in [18] and our results here validate our findings therein that the bioenergetic components we considered in this model constitute a basic set of oxidative processes for *H. salinarum*.

The limited agreement of our model with experimental data on oxygen consumption (Fig 5) provides an independent validation for our model. We did not explicitly incorporate oxygen consumption in the model, and only calculated oxygen consumption after the parameters were already estimated. Another independent validation of our model is its ability to model the phenomenon of constant sum of ΔΨ and 58ΔpH [4]; this property was not explicitly enforced in the model.

One shortcoming of the model is the inability to exhibit the correct concavity of the time courses for 58ΔpH and ΔΨ (Fig 4). Although we hypothesized that a possible explanation is the difference of the time dependence of the membrane potential used by other ion translocating processes between aerobic and anaerobic light conditions, it is also possible that other bioenergetics components, including other ion transporters could be necessary to correctly model concavity. This issue will be addressed in future work.

The shortcomings of the electrogenic model that we have mentioned above (the best parameter EG1 showed poor fit to ${\text{Na}}_{\text{i}}^{+}$ and ${\text{K}}_{\text{i}}^{+}$ data, no increase of pmf and increase of 58ΔpH, proton/sodium ratio <1) are most likely due to the inherent numerical difficulty in performing parameter estimation on the electrogenic model. The electrogenic model is 5 orders of magnitude more sensitive to the proton/sodium ratio than other model parameters. Hence, we do not make the conclusion at this point that an electroneutral model is able to fit the data better than the electrogenic model. What we have shown from the parameter sets we obtained (from both electrogenic and electroneutral models) is that the mathematical representation of the model has the correct network interactions to be consistent with experimental data. To answer the electrogenicity of the antiport using our model, data on membrane potential and ΔpH generation driven solely by respiration is needed, which is not within the scope of this study.

## Methods

### Model Formulation

In this section we provide the details of our derivation of the mathematical expressions of the fluxes in Fig 1 and Eqs (1) and (2).

#### Algebraic Variables

We describe the mathematical expressions for the algebraic variables in Table 1. We model a 1 ml suspension with 1 OD cells and we assume that the total concentration of proton, sodium and potassium ions are constant, which we denote respectively as H_tot_, K_tot_ and Na_tot_. Thus, given the individual cellular concentration of ${\text{H}}_{\text{i}}^{+}$, ${\text{K}}_{\text{i}}^{+}$, and ${\text{Na}}_{\text{i}}^{+}$, we compute the extracellular concentrations as

$$\begin{array}{ccc} \left[{\text{H}}_{\text{o}}^{+}\right] & = & \left({\text{H}}_{\text{tot}}-\left[{\text{H}}_{\text{i}}^{+}\right]a_{1}\right)/a_{2} \end{array}$$ (3)

$$\begin{array}{ccc} \left[{\text{K}}_{\text{o}}^{+}\right] & = & \left({\text{K}}_{\text{tot}}-\left[{\text{K}}_{\text{i}}^{+}\right]a_{1}\right)/a_{2} \end{array}$$ (4)

$$\begin{array}{ccc} \left[{\text{Na}}_{\text{o}}^{+}\right] & = & \left({\text{Na}}_{\text{tot}}-\left[{\text{Na}}_{\text{i}}^{+}\right]a_{1}\right)/a_{2}\;, \end{array}$$ (5)

where *a*_1_ is the total cellular volume of all cells in the medium, and *a*_2_ is extracellular volume in the medium. The values of *a*_1_ and *a*_2_ were obtained from Fig 6.

The proton motive force (pmf) is given by

$$\begin{array}{c} \text{pmf}=\frac{\Delta \mu_{{\text{H}}^{+}}}{\text{F}}=\Delta \Psi -\frac{\text{RT}}{\text{F}\;{\text{log}}_{10}\left(\text{e}\right)}\Delta \text{pH}\;. \end{array}$$ (6)

R is the gas constant, T is the temperature (in Kelvin), F is the Faraday constant and ΔΨ is the membrane potential (Table 5). The pH gradient is

$$\begin{array}{c} \Delta \text{pH}={\text{pH}}_{\text{out}}-{\text{pH}}_{\text{in}}=-\log_{10}\frac{\left[{\text{H}}_{\text{o}}^{+}\right]}{\left[{\text{H}}_{\text{i}}^{+}\right]}\;, \end{array}$$ (7)

and where we expressed the free energy to transfer a mole of ions from inside to outside of the membrane as

$$\begin{array}{c} \Delta \mu_{{\text{H}}^{+}}=F\Delta \Psi -\frac{\text{RT}}{\log_{10}\left(\text{e}\right)}\Delta \text{pH}\;. \end{array}$$ (8)

Thus, for a net proton charge of positive outside and negative inside, Δ*μ*_H^+^_ is positive and the free energy to transfer the protons from outside to inside the membrane is −Δ*μ*_H^+^_. We will also use this convention for the sign of the free energy in modeling the fluxes involving the (positively charged) sodium and potassium ions below. Moreover, we will adopt the convention that for fluxes through the membrane potential, a positive value is oriented outwards.

**Table 5:** Initial values of the model variables and constant total concentrations.

Initial Conditions			
Dependent Variables		Algebraic Variables	
[${\text{H}}_{\text{i}}^{+}$]	1.0000 × 10^−7^	[ADP]	1.7120 × 10^−3^
[ATP]	5.8800 × 10^−4^	pmf	1.0956 × 10^−1^
[${\text{K}}_{\text{i}}^{+}$]	2.3000	ΔpH	−5.0000 × 10^−1^
[${\text{Na}}_{\text{i}}^{+}$]	1.9000	ΔpK	2.0193
ΔΨ	8.0000 × 10^−2^	ΔpNa	−3.0103 × 10^−1^
[${\text{H}}_{\text{o}}^{+}$]	3.1623 × 10^−7^	Δ*μ*_H^+^_	1.0572 × 10^4^
[${\text{Na}}_{\text{o}}^{+}$]	3.8000	Δ*μ*_K^+^_	-3.8011 × 10^3^
[${\text{K}}_{\text{o}}^{+}$]	2.2000 × 10^−2^	Δ*μ*_Na^+^_	9.4368 × 10^3^
Total concentrations			
[H^+^]		3.1593 × 10^−7^ mol/liter	
[K^+^]		0.0251 mol/liter	
[Na^+^]		3.7974 mol/liter	
Other constants			
R (gas constant)		8.314462 joules/mol	
T temperature at 25°C		25 + 273 K	
F Faraday constant		96485 coul/mol	
*n*_*syn*_ H^+^/ATP		4	

These initial conditions were obtained from published values. The references and experimental conditions which yield these initial conditions are discussed in S1 File. See Table 1 for units.

The algebraic variable ΔpNa is given by

$$\begin{array}{c} \Delta \text{pNa}={\text{pNa}}_{\text{out}}-{\text{pNa}}_{\text{in}}=-\log_{10}\frac{\left[{\text{Na}}_{\text{o}}^{+}\right]}{\left[{\text{Na}}_{\text{i}}^{+}\right]}\;. \end{array}$$ (9)

For the electrogenic model, where the sodium ion extrusion is not balanced by proton intrusion, the free energy to transfer a mole of ions from inside to outside the membrane is included in the model, and it is given by

$$\begin{array}{c} \Delta \mu_{{\text{Na}}^{+}}=F\Delta \Psi -\frac{\text{RT}}{\log_{10}\left(\text{e}\right)}\Delta \text{pNa}\;. \end{array}$$ (10)

Similarly, for the potassium ions, the algebraic variables are given by

$$\begin{array}{c} \Delta \mu_{{\text{K}}^{+}}=F\Delta \Psi -\frac{\text{RT}}{\log_{10}\left(\text{e}\right)}\Delta \text{pK}\;, \end{array}$$ (11)

and

$$\begin{array}{c} \Delta \text{pK}={\text{pK}}_{\text{out}}-{\text{pK}}_{\text{in}}=-\log_{10}\frac{\left[{\text{K}}_{\text{o}}^{+}\right]}{\left[{\text{K}}_{\text{i}}^{+}\right]}\;. \end{array}$$ (12)

#### ATP Synthase Flux

*J*_*ATPS*_ denotes the inward flux of protons through the ATP synthase and the synthesis of ATP from ADP and Pi.

The ATP reaction is given by

$$\begin{array}{c} n_{\text{syn}}{\text{H}}_{\text{o}}^{+}+\text{ADP}+\text{Pi}⇌n_{\text{syn}}{\text{H}}_{\text{i}}^{+}+\text{ATP}\;. \end{array}$$

We are interested in ATP synthesis as the proton motive force is built by respiration. Therefore, the proton motive force remains above the point of reversibility of the ATP synthase and hence only the forward reaction (ATP synthesis) will be considered in our numerical simulations. A positive sign of the flux denotes the inward (or forward) flux of protons and the total sum of ATP and ADP is constant in the cell. The parameter *n*_syn_ denotes the stoichiometry H^+^/ATP. In archaea, values from 2 to 4 H^+^/ATP have been reported [29, 30] and we use the value 4 here. We did not consider the growth of the cells in the model due to the short duration of the bioenergetics experiments (at most one hour). Furthermore, due to the use of rich medium in which enough organic phosphate is available for the organism, utilization of cellular Pi for processes other than ATP synthesis are not considered. Thus, during the simulation time interval it is assumed that the concentration of Pi is constant. Since we consider “normal” cell conditions where Pi is in excess of ATP and ADP, the reaction ADP + Pi → ATP is not limited by Pi. The reaction flux is given by

$$\begin{array}{c} J_{ATPS}=\alpha_{ATPS}\times {\left[\text{ADP}\right]}^{\gamma_{ATPS:ADP}}\times \left[\text{Pi}\right]\times \left[\text{ATPS}\right]\times \text{pmf}\;. \end{array}$$ (13)

The flux expression indicates that ADP, Pi, the concentration of ATP synthase ([ATPS]), and the proton motive force (pmf) all affect the rate of production of ATP. The incorporation of the ATP synthase concentration is for model flexibility but for our purposes, the time duration of 1 hour is short enough so that the ATP synthase concentration is constant.

The parameter *α*_*ATPS*_ is the kinetic rate of the equation while the parameter *γ*_*ATPS*: *ADP*_ is the kinetic order of ADP. In this paper, we will denote all reaction kinetic rates by the symbol *α*, subscripted by the corresponding flux, and we will denote all kinetic orders of variable *X* in flux *F* by *γ*_*F*: *X*_.

By combining the constants in Eq (13), we can simplify the flux as

$$\begin{array}{c} J_{ATPS}=\beta_{ATPS}\times {\left[\text{ADP}\right]}^{\gamma_{ATPS:ADP}}\times \text{pmf}\;, \end{array}$$ (14)

where

$$\begin{array}{c} \beta_{ATPS}=\alpha_{ATPS}\times \left[\text{Pi}\right]\times \left[\text{ATPS}\right]\;. \end{array}$$ (15)

Note that *n*_syn_ does not appear in the flux expression above but it appears in the equations of variables that are affected by the ATP synthase (see Eqs (1) and (22)).

#### Electron Transport Chain Flux

The flux *J*_*ETCP*_ denotes the outward flux of protons through the ETC and is modeled as

$$\begin{array}{ccc} J_{ETCP} & = & \alpha_{ETCP}\times \left[\text{XH}\right]\times \left[\text{ETCP}\right]\times \left[{\text{O}}_{2}\right]\times e^{-F\frac{\gamma_{ETCP:pmf}\text{pmf}}{\text{RT}}}\;. \end{array}$$ (16)

The flux expression reflects the network in Fig 1 where the concentrations of XH, O_2_ and the proton pumping complexes in the electron transport chain (denoted by ETCP) all affect the rate of change of proton pumping. Since [XH], [O_2_] and [ETCP] are constant, we simplify the flux expression as

$$\begin{array}{c} J_{ETCP}=\beta_{ETCP}\times e^{-F\frac{\gamma_{ETCP:pmf}\times \text{pmf}}{\text{RT}}}\;, \end{array}$$ (17)

where *β*_*ETCP*_ = *α*_*ETCP*_ × [XH] × [O_2_] × [ETCP].

The impact of pmf on the ATP synthase flux at the scenario we are interested in (from low to maximal pmf) can be modeled by a simple proportionality of *J*_*ATPS*_ to pmf (Eq (14)). However, the relationship between *J*_*ETCP*_ and the pmf is more complex: *J*_*ETCP*_ builds up the pmf, but only up to a maximum value of around 300 mV. Thus, the pmf inhibits the ETC, and this inhibition has very fast dynamics. We modeled *J*_*ETCP*_ in a similar manner to our model of the catalytic cycle of the light-driven proton pump bacteriorhodopsin [28], in which an exponential factor was used to model inhibition. The respiratory proton pumps are influenced by the pmf in the same manner as bacteriorhodopsin, hence we used the same expression of the exponential factor as in [28] to model voltage dependence of the flux. Note that we used the symbol *γ* for the parameter inside the exponential factor (*γ*_*ETCP*: *pmf*_) even though this parameter is not technically a kinetic order.

#### Sodium-proton Antiporter Flux

*J*_*NaH*_ is the inward flux of protons and outward flux of sodium ions through the sodium-proton antiporter which is given by the reaction ${\text{H}}_{\text{o}}^{+}+{\text{Na}}_{\text{i}}^{+}\to {\text{H}}_{\text{i}}^{+}+{\text{Na}}_{\text{o}}^{+}$. The sodium-proton antiporter in *H. salinarum* is still not completely understood; it is possible that the antiporter is electroneutral (i.e., the number of protons and sodium ions are equal hence it does not use the membrane potential) or it could be electrogenic. For an electroneutral antiporter, the flux is given by

$$\begin{array}{c} J_{NaH}^{n}=\alpha_{NaH}\left(\Delta \text{pNa}-\Delta \text{pH}\right)\;. \end{array}$$ (18)

To model the electrogenic flux, we denote the ratio of protons per sodium ion as *n*_NaH_. The flux of the sodium-proton antiporter is then given by

$$\begin{array}{c} J_{NaH}^{e}=\alpha_{NaH}\left(\Delta \mu_{{\text{Na}}^{+}}-n_{\text{NaH}}\Delta \mu_{{\text{H}}^{+}}\right)\;. \end{array}$$ (19)

#### Potassium Uniport Flux

The flux *J*_*K*_ is given by

$$\begin{array}{c} J_{K}=\alpha_{K}\times \Delta \mu_{{\text{K}}^{+}}\;. \end{array}$$ (20)

#### ATP consumption rate

The rate of ATP use in the organism is modeled as

$$\begin{array}{c} J_{ATPuse}=\alpha_{ATPuse}\times [\text{ATP}]\;. \end{array}$$ (21)

The flux expression does not involve a kinetic order (*γ*_*ATPuse*: *ATP*_ = 1) because adding this kinetic order as a model parameter did not improve the fit (data not shown). Thus, we assumed its value to be equal to one in order to minimize the number of parameters. The only other kinetic order in our equations is in the ATP synthase flux, where the kinetic order *γ*_*ATPS*: *ADP*_ was necessary to fit the data.

We have thus presented the mathematical expressions for the fluxes in the model. An alternative approach is to use the Michaelis-Menten method for modeling enzyme kinetics. However, we chose the use of reaction rates and kinetic orders, since our modeling approach was motivated by the flexibility of the power-law type of models in biochemical systems [31].

#### Rate of Change of Membrane Potential

To model the rate of change of the membrane potential, we compute the current (of positive charges) produced by each flux which transports ions through the membrane. For a positive net flux, the membrane potential increases while it decreases for a negative net movement of ions. For an electroneutral sodium-proton antiport, the net charge of the ions translocated is zero hence fluxes due to the antiport are not included in the rate of change of the membrane potential. In this case, the fluxes affecting the membrane potential are *J*_*ATPS*_, *J*_*ETCP*_ and *J*_*K*_. The current produced by these fluxes is computed by multiplying the fluxes with the volume of the cell, Avogadro’s number (6.022140857 × 10^23^ mol^−1^) and by the elementary charge (1.602176565 × 10^−19^ coul). The product of these three quantities will be denoted as *k*_1_ and by denoting the number of protons involved in the phosphorylation of one molecule of ADP by the ATP synthase by *n*_syn_, then the rate of change of the membrane potential is given by

$$\begin{array}{c} \frac{d\Delta \Psi }{dt}=\frac{\alpha_{mempot}k_{1}}{C_{m}}(J_{ETCP}-n_{\text{syn}}J_{ATPS}-J_{K})\;, \end{array}$$ (22)

where *C*_*m*_ is the cell membrane capacitance. The estimation of the value of *C*_*m*_ is given in S1 File. For an electrogenic system, the rate of change of the membrane potential now includes the flux from the antiport

$$\begin{array}{c} \frac{d\Delta \Psi }{dt}=\frac{\alpha_{mempot}k_{1}}{C_{m}}(J_{ETCP}-n_{\text{syn}}J_{ATPS}-J_{K}-\left(n_{\text{NaH}}-1\right)J_{NaH}^{e})\;. \end{array}$$ (23)

We lump together the three parameters *α*_*mempot*_, *k*_1_ and *C*_*m*_ into one parameter

$$\begin{array}{c} \beta_{mempot}=\frac{\alpha_{mempot}k_{1}}{C_{m}}\;. \end{array}$$ (24)

### Parameter Estimation

To estimate the values of the unknown parameters, we formulated a cost function that, for a given set of parameter values, measures the difference between model output and experimental data. The experimental data taken from literature consists of time-course sodium and potassium concentration [3] and steady state concentration of ATP [1] and pmf [4, 5]. We used the steady state values for ATP concentration and the pmf since these variables rapidly reached their steady-states (within milliseconds for pmf [4, 5] and within seconds for ATP [1]). On the other hand, the sodium and potassium concentrations took about an hour to reach steady state [3], and thus their dynamics dominated the time range of our simulations.

The optimal parameter values were obtained by numerically minimizing the cost function

$$\begin{array}{ccc} \min_{p\in R^{Np}}J\left(p\right) & = & \frac{1}{N_{K}}\sqrt{\frac{\sum_{k=1}^{N_{K}}{\left({\left[{\text{K}}_{\text{i}}^{+}\right]}^{data}\left(t_{k}\right)-{\left[{\text{K}}_{\text{i}}^{+}\right]}^{model}\left(p;t_{k}\right)\right)}^{2}}{\sum_{k=1}^{N_{K}}{\left[{\text{K}}_{\text{i}}^{+}\right]}^{data}{\left(t_{k}\right)}^{2}}}\\ & + & \frac{1}{N_{Na}}\sqrt{\frac{\sum_{k=1}^{N_{Na}}{\left({\left[{\text{Na}}_{\text{i}}^{+}\right]}^{data}\left(t_{k}\right)-{\left[{\text{Na}}_{\text{i}}^{+}\right]}^{model}\left(p;t_{k}\right)\right)}^{2}}{\sum_{k=1}^{N_{Na}}{\left[{\text{Na}}_{\text{i}}^{+}\right]}^{data}{\left(t_{k}\right)}^{2}}}\\ & + & \frac{w_{ATP}}{{\left[ATP\right]}^{data}\left(t_{final}\right)}|{\left[ATP\right]}^{data}\left(t_{final}\right)-{\left[ATP\right]}^{model}\left(t_{final}\right)|\\ & + & \frac{w_{pmf}}{pmf^{data}\left(t_{final}\right)}|pmf^{data}\left(t_{final}\right)-pmf^{model}\left(t_{final}\right)|\;, \end{array}$$ (25)

subject to the ODE model Eqs (1) or (2),

where *p* is the vector of parameter values with length *Np* (*Np* = 8 for the electroneutral model and *Np* = 9 for the electrogenic model), *t*_*k*_ are time points where data were measured, and *N*_*K*_ and *N*_*N*_*a*__ are the number of data points for $\left[{\text{K}}_{\text{i}}^{+}\right]$ and $\left[{\text{Na}}_{\text{i}}^{+}\right]$, respectively. *t*_*final*_ is the time point where steady state ATP and pmf were measured.

Each term in Eq (25) measures the difference between one set of data and the model output, and each term was normalized. Normalization was performed in order to scale the values. Thus the cost function calculates the relative error between the model and the data. We used the weights *w*_*ATP*_ and *w*_*pmf*_ to give more importance to fitting the steady state ATP concentration and to allow more flexibility in fitting the pmf (*w*_*ATP*_ = 10, *w*_*pmf*_ = 0.5). These weights do not affect the theoretical optimal solution of the cost function. However, in practice we found that using these weights helped us obtain better convergence with the numerical methods and thus we used these values in all our parameter estimation calculations in the paper. The values of the experimental data are given in Table 6.

**Table 6:** Experimental data used in the cost function (Eq (25)).

steady state ATP		3.7 mmol/kg cell water
steady state pmf		280 mV
time (min)	${\text{K}}_{\text{i}}^{+}$ (mmol/liter)	${\text{Na}}_{\text{i}}^{+}$ (mmol/liter)
0	2304.7	1851.6
4.8	2437.5	1695.3
10	2601.6	1718.8
20	2828.1	1492.2
40	2828.1	NA
60	2937.5	1296.9

All values were taken from literature: ATP [1], pmf [4] Na^+^ and K^+^ [3].

To evaluate the model at the data time-points, we numerically solved the ODE using Matlab’s stiff solver ode15s, providing the solver with initial conditions (Tables 1 and 5). We used a relative tolerance of 1 × 10^−6^ and an absolute tolerance of 1 × 10^−8^ in numerical ODE calculations. To minimize the cost function Eq (25), we used two optimization algorithms: Matlab’s fminsearch function (which is the simplex-based Nelder Mead algorithm) and a Matlab implementation of the Simulated Annealing algorithm (J. Vandekerckhove, Matlab Central). For the Nelder Mead algorithm, we used the tolerance of 1 × 10^−6^ for TolCon, TolFun, and TolX. For the Simulated Annealing algorithm, we used a tolerance of 1 × 10^−6^ for the stop temperature.

We separately solved for the optimal parameters of the electrogenic and electroneutral models. To estimate the parameters of the electroneutral model, we supplied an initial guess of a vector of ones to the simulated annealing algorithm (Table 2, Set EN1). However, the resulting model did not fit well the experimental data on $\left[{\text{K}}_{\text{i}}^{+}\right]$ and $\left[{\text{Na}}_{\text{i}}^{+}\right]$. We therefore performed manual fitting by varying the parameter values ourselves, and found another set of parameters that yielded a model output which qualitatively fitted the internal potassium and sodium concentrations better than set EN1 (Table 2, Set EN2). We then used the manually obtained parameters as the initial parameter guess for another round of optimization using Nelder-Mead (Table 2, Set EN3) and Simulated Annealing (Table 2, Set EN4). Parameter set EN4 produced a model with a worse fit to the data and was dropped. We performed one final round of optimization by using set EN3 as the initial guess for Simulated Annealing (Table 2, Set EN5). EN5 is our best set of parameters for the electroneutral model.

Note that the best set of parameters that we chose (set EN5) did not yield the lowest value for the cost function (Table 2). We had to rely on our own manual evaluation of the model output and discarded those that qualitatively did not reflect the data. This practice, although not ideal, is typically done when modeling biochemical systems where modelers follow an iterative process that involves manual intervention when choosing parameter values or network connectivity [32].

We performed the same strategy to obtain a set of parameter values for the electrogenic model. The parameter sets obtained in Table 3 were obtained using the same algorithms as in the electroneutral model (e.g., EG1 was obtained using the same algorithms as EN1), except that in this case we considered an additional parameter which is the ratio of protons to sodium ions. Our best set of parameters for the electrogenic model was given by set EG1, which was obtained using Simulated Annealing with an initial guess of a vector of ones. As in the electroneutral case, the set of parameters we chose did not yield the lowest value for the cost function (Table 3).

All plots of the model using the parameter sets in Tables 2 and 3 are given in S1 File. We also provide the matlab code we used for parameter estimation (S1 Matlab Code).

### Parameter Sensitivity Analysis

For a given model output at a certain time instance, we want to quantify the model sensitivity to minute changes in the values of a parameter. Consider variable *X*_*i*_ and denote the model output at *X*_*i*_ evaluated at time *t*_*k*_ by *X*_*i*_(*t*_*k*_). Then the sensitivity of *X*_*i*_ to infinitesimal changes in parameter *p*_*j*_ is given by

$$\begin{array}{c} {\frac{\partial X_{i}\left(t\right)}{\partial p_{j}}|}_{t=t_{k}}\;. \end{array}$$

Since the model variables and model parameters have various units, the sensitivity is normalized and denoted as *S*_*ij*_(*t*_*k*_)

$$\begin{array}{c} S_{ij}\left(t_{k}\right)={\frac{p_{j}\partial X_{i}\left(t\right)}{X_{i}\left(t_{k}\right)\partial p_{j}}|}_{t=t_{k}}={\frac{\partial \log(X_{i}\left(t_{k}\right))}{\partial \log(p_{j})}|}_{t=t_{k}}\;. \end{array}$$ (26)

The model and parameter values *X*_*i*_(*t*_*k*_) (*i* = 1, …, *n*;*t*_*k*_ = 1, …, *N*_*t*_) and *p*_*j*_ (*j* = 1, …, *m*) are denoted as the nominal values. Let us denote the *N*_*t*_ × *m* sensitivity matrix by **S**_*i*_, then the Fisher Information Matrix is given by

$$\begin{array}{c} \text{FIM}=\sum_{i=1}^{n}S_{i}^{T}Q_{i}^{-1}S_{i}\;. \end{array}$$

The matrix **Q**_*i*_ is the measurement error covariance matrix. Here we assume that **Q**_*i*_ is a diagonal matrix with elements $\sigma_{i,k}^{2}$, where *σ*_*i*,*k*_ = *ϵ*_1_ *X*_*i*_(*t*_*k*_)+*ϵ*_2_, where *ϵ*_1_ and *ϵ*_2_ are relative and absolute errors. The FIM consolidates the parameter sensitivities while accounting for the noise in measurements [33]. The sensitivity of the model to parameter *p*_*j*_, over all time points is calculated using the diagonal of the FIM

$$\begin{array}{c} r_{j}=\sqrt{\sum_{i=1}^{n}\sum_{k=1}^{Nt}{\left(\frac{p_{j}}{\sigma_{i,k}}\frac{\partial X_{i}\left(t_{k}\right)}{\partial p_{j}}\right)}^{2}}\;. \end{array}$$ (27)

To compute the sensitivities, we first derived analytical expressions of the derivatives of the ODE model with respect to each parameter. This resulted in a system of *n* × *m* “sensitivity” equations (*n* is the number of variables and *m* is the number of parameters). We then numerically solved the ODE model and the sensitivity equations simultaneously (*n*+*n* × *m* differential equations). The ODE was numerically integrated using Matlab’s stiff ODE solver and evaluated at *Nt* discrete time points (*t*_1_, …, *t*_*k*_, …*t*_*Nt*_). The values of the relative and absolute errors used as tolerances for the ODE solver were 1 × 10^−8^ and 1 × 10^−10^, respectively, which we also used for the values of *ϵ*_1_ and *ϵ*_2_ in the FIM.

## Supporting Information

S1 File Supplementary Text and Figures. The supplementary text contains details about the derivation of cellular constants, medium constants and initial conditions. The file also contains parameter sensitivity results and model output using all parameter sets.
(PDF)

S1 Matlab Code Matlab code for parameter estimation and for plotting results. We provide a set of scripts that can be used to plot the model output using the parameter sets in Tables 2 and 3. An example of parameter estimation to obtain parameter set EN3 is provided. Also provided are scripts that can be used for converting experimental measurements into different units.
(ZIP)

## References

[1] Hartmann R, Oesterhelt D. Bacteriorhodopsin-mediated photophosphorylation in *Halobacterium halobium*. Eur J Biochem. 1977;77:325–335. 10.1111/j.1432-1033.1977.tb11671.x 19249

[2] Hartmann R, Sickinger H, Oesterhelt D. Anaerobic growth of halobacteria. Proc Natl Acad Sci USA. 1980;77:3821–3825. 10.1073/pnas.77.7.3821 6933439 PMC349718

[3] Wagner G, Hartmann R, Oesterhelt D. Potassium uniport and ATP synthesis in *Halobacterium halobium*. Eur J Biochem. 1978;89:169–179. 10.1111/j.1432-1033.1978.tb20909.x 29755

[4] Michel H, Oesterhelt D. Electrochemical proton gradient across the cell membrane of *Halobacterium halobium*: effect of N,N’-Dicyclohexylcarbodiimide, relation to intracellular adenosine triphosphate, adenosine diphosphate, and phosphate concentration, and influence of the potassium gradient. Biochemistry. 1980;19(20):4607–4614. 7426619 10.1021/bi00561a011

[5] Michel H, Oesterhelt D. Electrochemical proton gradient across the cell membrane of *Halobacterium halobium*: comparison of light-induced increase with the increase of intracellular adenosine triphosphate under steady-state illumination. Biochemistry. 1980;19(20):4615–4619. 10.1021/bi00561a012 7426620

[6] Danon A, Stoeckenius W. Phosphorylation in *Halobacterium halobium*. Proc Natl Acad Sci USA. 1974;71:1234–1238. 10.1073/pnas.71.4.1234 4524635 PMC388199

[7] Müller J, DasSarma S. Genomic analysis of anaerobic respiration in the archaeon Halobacterium sp. strain NRC-1: dimethyl sulfoxide and trimethylamine N-oxide as terminal electron acceptors. J Bacteriol. 2005;187:1659–1667. 10.1128/JB.187.5.1659-1667.2005 15716436 PMC1064022

[8] Dundas IE, Halvorson HO. Arginine metabolism in *Halobacterium salinarum*, an obligately halophilic nacterium. J Bacteriol. 1966;91:113–119. 5903088 10.1128/jb.91.1.113-119.1966 PMC315918

[9] Gonzalez O, Gronau S, Pfeiffer F, Mendoza E, Zimmer R, Oesterhelt D. Systems analysis of bioenergetics and growth of the extreme halophile *Halobacterium salinarum*. PLoS Comput Biol. 2009;5(4):e1000332 10.1371/journal.pcbi.1000332 19401785 PMC2674319

[10] Sreeramulu K, Schmidt CL, Schäfer G, Anemüller S. Studies of the electron transport chain of the euryarcheon *Halobacterium salinarum*: indications for a Type II NADH dehydrogenase and a complex III analog. J Bioenerg Biomembr. 1998;30(5):443–453. 10.1023/A:1020538129400 9932647

[11] Pfeiffer F, Schuster SC, Broicher A, Falb M, Palm P, Rodewald K, et al Evolution in the laboratory: the genome of *Halobacterium salinarum* strain R1 compared to that of strain NRC-1. Genomics. 2008;91(4):335–346. 10.1016/j.ygeno.2008.01.001 18313895

[12] Beard DA. A biophysical model of the mitochondrial respiratory system and oxidative phosphorylation. PLoS Comput Biol. 2005;1(4):252–264. 10.1371/journal.pcbi.0010036 PMC1201326 16163394

[13] Korzeniewski B, Froncisz W. Theoretical studies on the control of oxidative phosphorylation system. Biochim Biophys Acta. 1992;1102:67–75. 10.1016/0005-2728(92)90066-B 1324730

[14] Korzeniewski B. Simulation of state 4 → state 3 transition in isolated mitochondria. Biophys Chem. 1996;57:143–153. 10.1016/0301-4622(95)00076-7 17023337

[15] Magnus G, Keizer J. Model of *β*-cell mitochondrial calcium handling and electrical activity. I. Cytoplasmic variables. Am J Physiol (Cell Physiol 43). 1998;274:C1158–C1173. 10.1152/ajpcell.1998.274.4.C1158 9575813

[16] Bertram R, Pedersen MG, Luciani DS, Sherman A. A simplified model for mitochondrial ATP production. J Theor Biol. 2006;243:575–586. 10.1016/j.jtbi.2006.07.019 16945388

[17] Klamt S, Grammel H, Straube R, Ghosh R, Gilles E. Modeling the electron transport chain of purple non-sulfur bacteria. Mol Syst Biol. 2008;4:156 10.1038/msb4100191 18197174 PMC2238716

[18] del Rosario RCH, Mendoza ER, Oesterhelt D. Modelling the bioenergetics of *Halobacterium salinarum* with Petri nets. J Comput Theor Nanosci. 2009;6(8):1965–1976. 10.1166/jctn.2009.1252

[19] Lanyi JK, Silverman MP. Gating effects in *Halobacterium halobium* membrane transport. J Biol Chem. 1979;254(11):4750–4755. 35540

[20] Murakami N, Konishi T. Cooperative regulation of the NA^+^/H^+^-antiporter in *Halobacterium halobium* by ΔpH and Δ*φ*. Arch Biochem Biophys. 1990;281(1):13–20. 10.1016/0003-9861(90)90406-O 2166477

[21] Albers SV, de Vossenberg JLCMV, Driessen AJM, Konings WN. Bioenergetics and solute uptake under extreme conditions. Extremophiles. 2001;5:285–294. 11699642 10.1007/s007920100214

[22] Michel H, Oesterhelt D. Light-induced changes in the pH gradient and membrane potential in *Halobacterium halobium*. FEBS Lett. 1976;65:175–178. 10.1016/0014-5793(76)80473-5 6333

[23] Hartmann R, Sickinger HD, Oesterhelt D. Quantitative aspects of energy conversion in halobacteria. FEBS Lett. 1977;82:1–6. 10.1016/0014-5793(77)80873-9 21098

[24] Oesterhelt D, Krippahl G. Light inihibition of respiration in *Halobacterium halobium*. FEBS Lett. 1973;32:72–76. 10.1016/0014-5793(73)80339-4 4747602

[25] Koch MK, Oesterhelt D. MpcT is the transducer for membrane potential changes in *Halobacterium salinarum*. Mol Microbiol. 2005;55(6):1681–1694. 10.1111/j.1365-2958.2005.04516.x 15752193

[26] Koch M. Investigations on halobacterial transducers with respect to membrane potential sensing and adaptive methylation. Munich, Germany: Ludwig-Maximilians-Universitaet Muenchen; 2005.

[27] del Rosario RCH, Staudinger WF, Streif S, Pfeiffer F, Mendoza E, Oesterhelt D. Modelling the CheY^*D*10*K*,*Y*100*W*^ *Halobacterium salinarun* mutant: sensitivity analysis allows choice of parameters to be modified in the phototaxis model. IET Syst Biol. 2007;1(4):207–221. 10.1049/iet-syb:20070007 17708428

[28] del Rosario RCH, Oppawsky C, Tittor J, Oesterhelt D. Modelling the membrane potential generation of bacteriorhodopsin. Math Biosci. 2010;225:68–80. 10.1016/j.mbs.2010.02.002 20188746

[29] Schäfer G, Engelhard M, Müller V. Bioenergetics of the archaea. Microbiol Mol Biol Rev. 1999;63(3):570–620. 10477309 10.1128/mmbr.63.3.570-620.1999 PMC103747

[30] Oren A. Bioenergetic aspects of halophilism. Microbiol Mol Biol Rev. 1999;63(2):334–348. 10357854 10.1128/mmbr.63.2.334-348.1999 PMC98969

[31] Voit EO. Computational Analysis of Biochemical Systems: A Practical guide for Biochemists and Molecular Biologists. Cambridge University Press; 2000.

[32] Chou IC, Voit EO. Recent Developments in Parameter Estimation and Structure Identification of Biochemical and Genomic Systems. Math Biosci. 2009;219(2):57–83. 10.1016/j.mbs.2009.03.002 19327372 PMC2693292

[33] Varma A, Morbidelli M, Wu H. Parameteric Sensitivity in Chemical Systems. Cambridge, U.K: Cambridge University Press; 1999.

