# Supplementary material for: Model Construction and Analysis of Respiration in Halobacterium salinarum
Source: PLoS One. 2016 Mar 24;11(3):e0151839. doi: 10.1371/journal.pone.0151839 (PMC4806987; doi:10.1371/journal.pone.0151839)
Supplement: S1 File — The supplementary text contains details about the derivation of cellular constants, medium constants and initial conditions. The file also contains parameter sensitivity results and model output using all parameter sets. (PDF) [file pone.0151839.s001.pdf]

Supporting Information – S1 File

Model Construction and Analysis of Respiration in *Halobacterium salinarum*

Cherryl O. Talaue[1], Ricardo C.H. del Rosario[1,2,¤,*], Friedhelm Pfeiffer[2], Eduardo R. Mendoza[1,2], Dieter Oesterhelt[2]

1 Institute of Mathematics, University of the Philippines, Diliman, Quezon City, Philippines
2 Max Planck Institute of Biochemistry, Department of Membrane Biochemistry, Martinsried, Germany

¤Current Address: Broad Institute of Harvard and MIT, Cambridge, Massachusetts, USA
*** rcdelros@broadinstitute.org**

Cellular Constants, Medium Constants and Initial Conditions

In this section, we discuss the details on the estimation of the constant values of that were used in the model. Experimental data was measured either in a suspension (e.g. 1 mL suspension at 1 OD), or at different cellular concentration units, hence we needed to harmonize the quantities into a single system of units.

First we provide the details for the values in Fig 6, where most values were taken from [1–4]. A 1 mL suspension at 1 OD (100 Klett), contains 1.81 μL cell pellet and 1.36×10^9 halobacterial cells. The number of cells measured by different experiments range from 1.2×10^9 to 2×10^9, and we chose 1.36×10^9 to simplify calculations as this value will yield an individual cell volume of 1 fL (see below). The 1.81 μL cell pellet consists of 0.47 μL cellular organic material, 0.45 μL cellular basal salt and 0.89 μL inter-cellular basal salt (Fig 6A). The total cell volume within the cell pellet is 1.36 μL (1.36 = 0.47 + 0.89). This total cell volume contains 0.8 μL water. Using a density of 1.2 mg/mL for the cells, we obtain a total cell mass of 1.63 μg which contains 0.8 μg water. Assuming a protein content of 0.4 mg per 1OD 1 ml [1,2], then the cellular mass contains 0.4 μg protein. The remaining components by mass are 0.24 μg salt and 0.19 μg non-protein (Fig 6B). To obtain the mass of each cell, we divide the total cellular mass by 1.36×10^9 (Fig 6C). We consider a cylindrical cell with diameter 0.5 μm and length 5 μm. This yields an individual cell volume of 1 fL with a surface area of 8.13 μm^2. To obtain the other values in Fig 6D, we assume a cell membrane thickness of 60 angstroms, the membrane consists of 50% lipids and 50% protein and that a lipid molecule covers 65 angstrom^2.

The electric cell membrane capacitance was computed from $C_m = e_0 e_r S/b$, where $e_o = 8.85 \times 10^{-12}$ F $\cdot$ m^{-1} and $e_r = 3$. From Fig 6 of the main text, the surface area is $S = 8.13 \times 10^{-12}$ m^2, the membrane thickness is $b = 60$ angstrom, and substituting in these values in the formula for C_m yields $C_m = 3.60 \times 10^{-14}$ F. This is comparable to the value $C_m = 4.45 \times 10^{-14}$ F in [5] where the membrane thickness of $b = 60$ angstrom was also used. The slight difference was from the surface area computation.

To compute the initial conditions, i.e., the values of the model variables at $t = 0$, we use the following experimental data. The pH of the medium before the start of the simulation is 6.5 and the internal pH is 7.0. The initial ΔpH thus contributes approximately 0.5×60 or 30 mV to the pmf. The initial membrane potential is 80 mV [6]. The potassium ion gradient is large, about 100 fold, and from [7, Fig 1], we obtain the initial value of 2.3 mol/kg water for K_i^+ and 0.02

mol/kg water for K_o^+. The salt gradient is about two-fold and from [7, Fig 1], we obtain an initial internal sodium concentration of 1.9 mol/kg water and a medium salinity of 3.8 mol/kg water. For ATP, we use the value of 0.588 mmol/liter from Fig 3 in [4] where intracellular concentrations of ATP incubated for one hour in the dark was determined for different external pH levels.

The intracellular levels of inorganic phosphate in an exponentially growing culture supplied with Pi was measured in [8] to be 33.5 ± 3.7 mM and we used the value 35 mM here. Note that intracellular inorganic phosphate was also measured in [9] to be Pi = 52 mmol/kg water and using our unit conversion values in Fig 6 of the main text and the conversion scripts (S1 Matlab code), this value was converted to 30.6 mM, which is in agreement with [8].

The experimental setup in [10] involved 3 ml suspension with 1.5 OD at 25° C, 25% salinity and 760 mmHg pressure. Its O_2 solubility was 7.04 mg/liter which yields 6.60×10^{-7} moles O_2. The medium contains $1.5 \times (1.36 \times 10^9) \times 3$ cells yielding an initial (O_2 concentration at blocked respiration) cellular oxygen concentration of 0.108 mol/liter (Table 1 of main text).

The constant sum of ATP and ADP in the cell was obtained from the maximum ATP of 3.9 mmol/kg water in [7, Fig 2]. Using the cell parameters in Fig 6 of the main text and the conversion scripts (S1 Matlab code), we obtained the value of 2.3×10^{-1} mol/liter. This value is consistent with the maximum ATP value of 7.0 nmol/mg protein in [4] (7.0 nmol/mg protein was converted to $2.\times10^{-1}$ mol/liter using our conversion scripts (S1 Matlab code)).

XH was taken to be 10 mmol/kg water corresponding to 5.88×10^{-3} mol/liter, which is less than cellular Pi (Table 1 of the main text). ATP synthase concentration was computed from assuming 3×10^4 copies in the cell (under illumination, bacteriorhodopsin molecules range from about 2×10^5 to 4×10^5) while the combined concentration of proton pumps in the respiratory chain was computed from assuming 4×10^4 copies.

Determination of consumed oxygen of each individual cell from data in Oesterhelt and Krippahl (1973)

The consumed oxygen of a three 3 mL suspension with 1.5 OD was measured in Oesterhelt and Krippahl (1973) [10] as 8 μM (or 0.024 μ moles) in 48 minutes. We estimated the corresponding consumed oxygen of each individual cell as follows. From Fig 6 of the main text, a 1 OD 1 mL suspension contains 1.36×10^9 cells hence the total number of cells in [10] is approximated by $1.5\times(1.36 \times 10^9) \times 3$. Thus, the number of moles of oxygen consumed by each cell during 48 mins is 3.9216×10^{-12} μmoles $(0.024/(1.5 \times (1.36 \times 10^9) \times 3)$. By dividing this amount by the cell water volume (0.59 fL; Fig 6 of main text), we obtain the desired concentration which is 6.6315 mol/liter. The amount of consumed oxygen in [10] was linear with time and thus in the figures, we plotted consumed oxygen as a line passing through origin and the point (x=48 minutes, y=6.6315 mol/liter).

Table A. Sensitivities of the electroneutral parameters. The parameter sets correspond to those in Table 2 of the main text.

	FIM Diagonal Value				
Parameter	EN1	EN2	EN3	EN4	EN5
β_{ATPS}	3.7686×10^{14}	1.7376×10^{15}	2.2120×10^{15}	2.4618×10^{15}	2.3424×10^{15}
$\gamma_{ATPS,ADP}$	1.7817×10^{15}	2.5570×10^{15}	2.4357×10^{15}	8.2935×10^{15}	3.6532×10^{15}
β_{ETCP}	3.7134×10^{14}	1.7605×10^{15}	2.2354×10^{15}	2.4858×10^{15}	2.3605×10^{15}
$\gamma_{ETCP,pmf}$	1.6775×10^{15}	3.7086×10^{15}	4.3871×10^{15}	6.3458×10^{15}	5.9534×10^{15}
α_{NaH}	2.4791×10^{12}	4.4005×10^{12}	4.1086×10^{12}	4.5423×10^{12}	2.7487×10^{12}
α_{K}	1.4523×10^{11}	2.4624×10^{13}	2.5215×10^{13}	2.8522×10^{13}	2.0286×10^{13}
α_{ATPuse}	7.5653×10^{13}	5.2141×10^{13}	6.3936×10^{13}	7.3767×10^{13}	6.4708×10^{13}
β_{mempot}	8.0642×10^{09}	1.0870×10^{10}	1.1043×10^{10}	8.7555×10^{09}	8.1855×10^{09}

Table B. Sensitivities of the electrogenic parameters. The parameter sets correspond to those in Table 3 of the main text.

	FIM Diagonal Value				
Parameter	EG1	EG2	EG3	EG4	EG5
β_{ATPS}	4.2155×10^{14}	1.7516×10^{15}	2.4174×10^{15}	1.3462×10^{15}	2.4779×10^{15}
$\gamma_{ATPS,ADP}$	1.8098×10^{15}	2.5663×10^{15}	2.3348×10^{15}	2.4704×10^{15}	4.5989×10^{15}
β_{ETCP}	4.1357×10^{14}	1.8008×10^{15}	2.4531×10^{15}	1.3930×10^{15}	2.4900×10^{15}
$\gamma_{ETCP,pmf}$	1.9887×10^{15}	3.7634×10^{15}	4.7875×10^{15}	2.0177×10^{15}	2.2971×10^{15}
α_{NaH}	7.0068×10^{11}	2.8814×10^{13}	1.5047×10^{13}	2.7018×10^{13}	4.9582×10^{12}
α_{K}	4.4818×10^{10}	2.7309×10^{13}	2.4826×10^{13}	2.8425×10^{13}	1.0072×10^{13}
α_{ATPuse}	8.4650×10^{13}	5.2141×10^{13}	7.1945×10^{13}	4.0680×10^{13}	6.7111×10^{13}
β_{mempot}	1.7694×10^{10}	5.4644×10^{13}	2.6779×10^{13}	5.0912×10^{13}	9.1266×10^{12}
n_{NaH}	5.4802×10^{20}	4.3683×10^{22}	2.7103×10^{22}	3.9133×10^{22}	1.0450×10^{22}

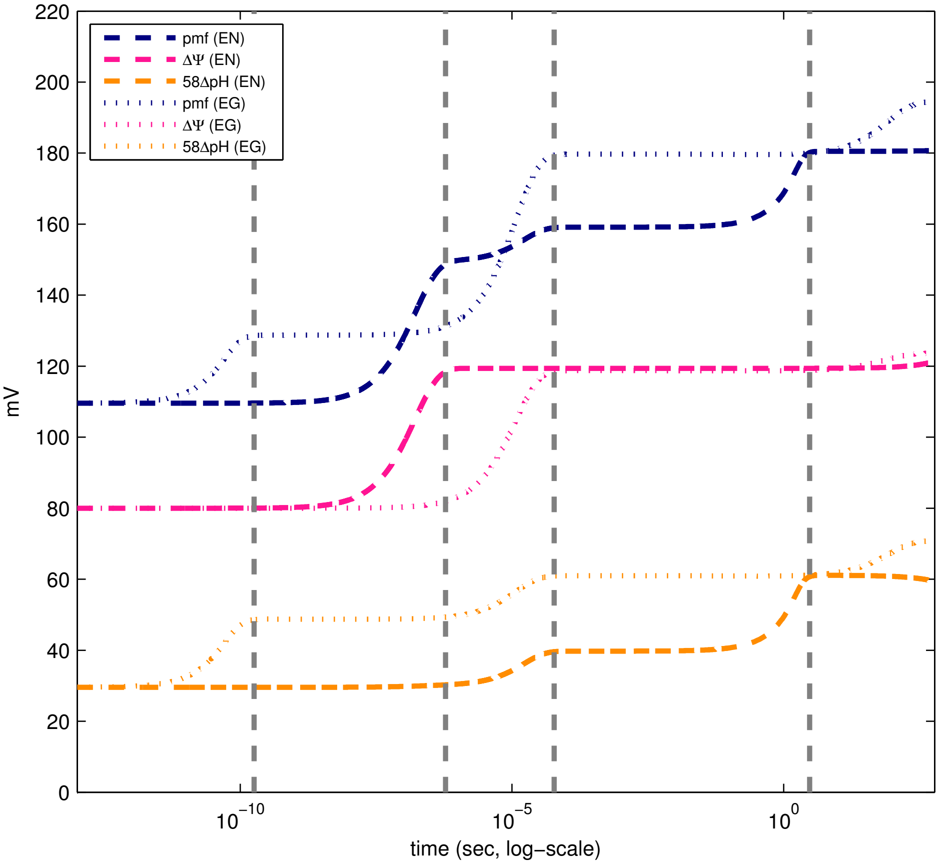

Fig A. Fig 4 plotted in logarithmic scale up to 10 minutes. The electroneutral model has jumps at t=6×10^{-7}, 6×10^{-5} and 5.4 seconds. The values of 58ΔpH decreased starting at 5.4 seconds, while ΔΨ increased (see also Fig 4). The electrogenic model has jumps at t=1.8×10^{-10} and t=6×10^{-5}. After 5.4 seconds, it increased monotonically up to the maximal pmf (Fig 4).

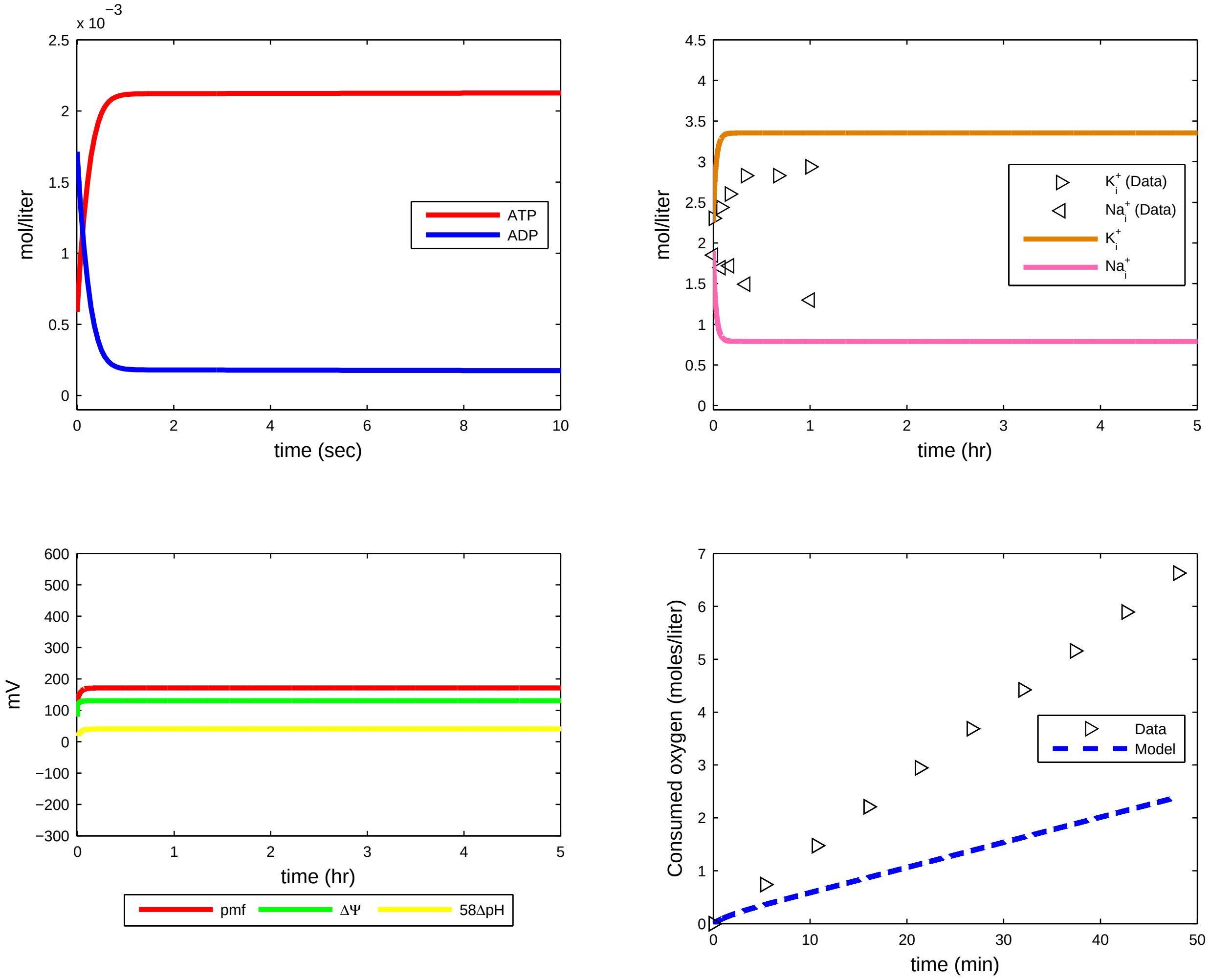

Fig B. Electroneutral model output using parameter set EN1 (Table 2).

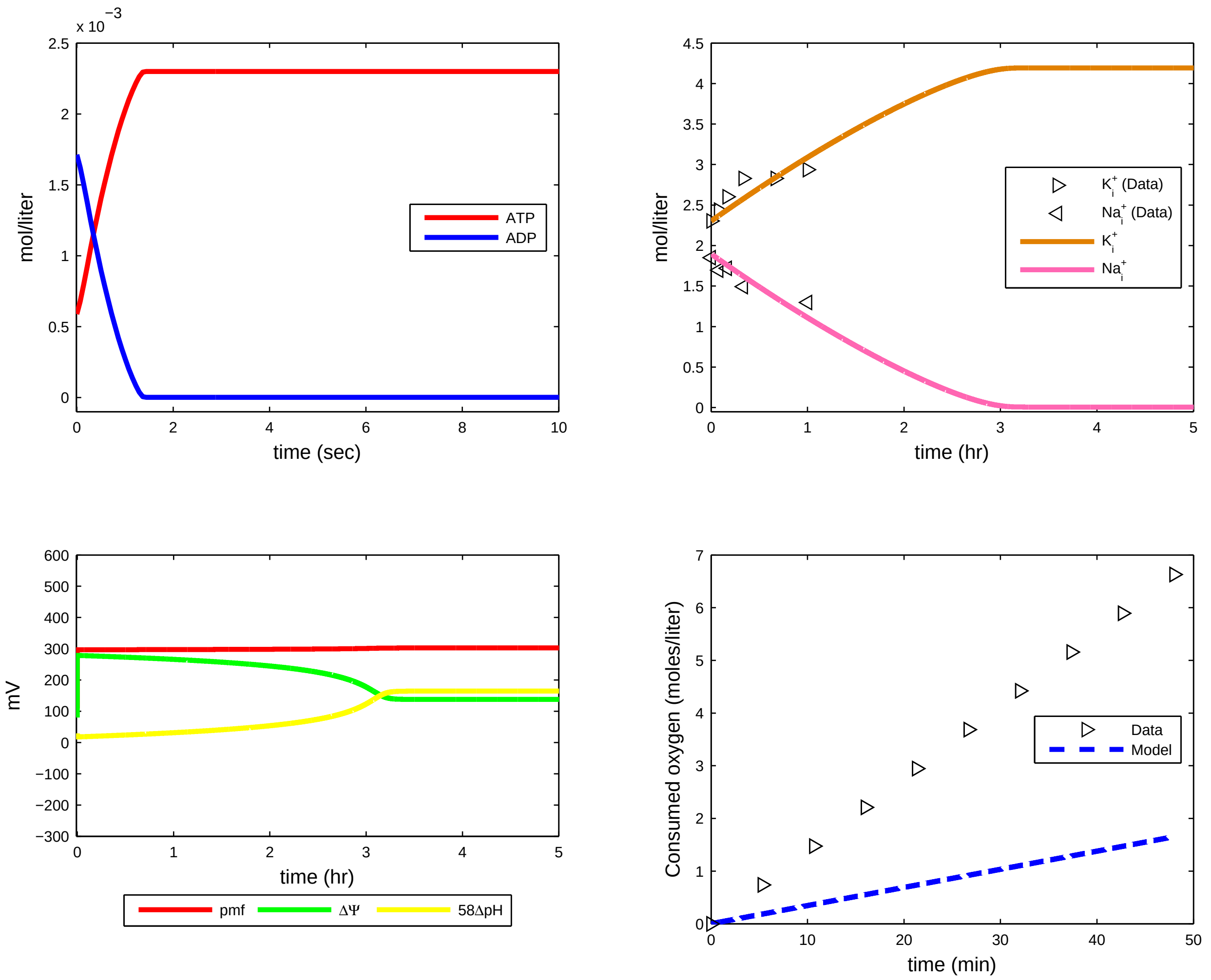

Fig C. Electroneutral model output using parameter set EN2 (Table 2).

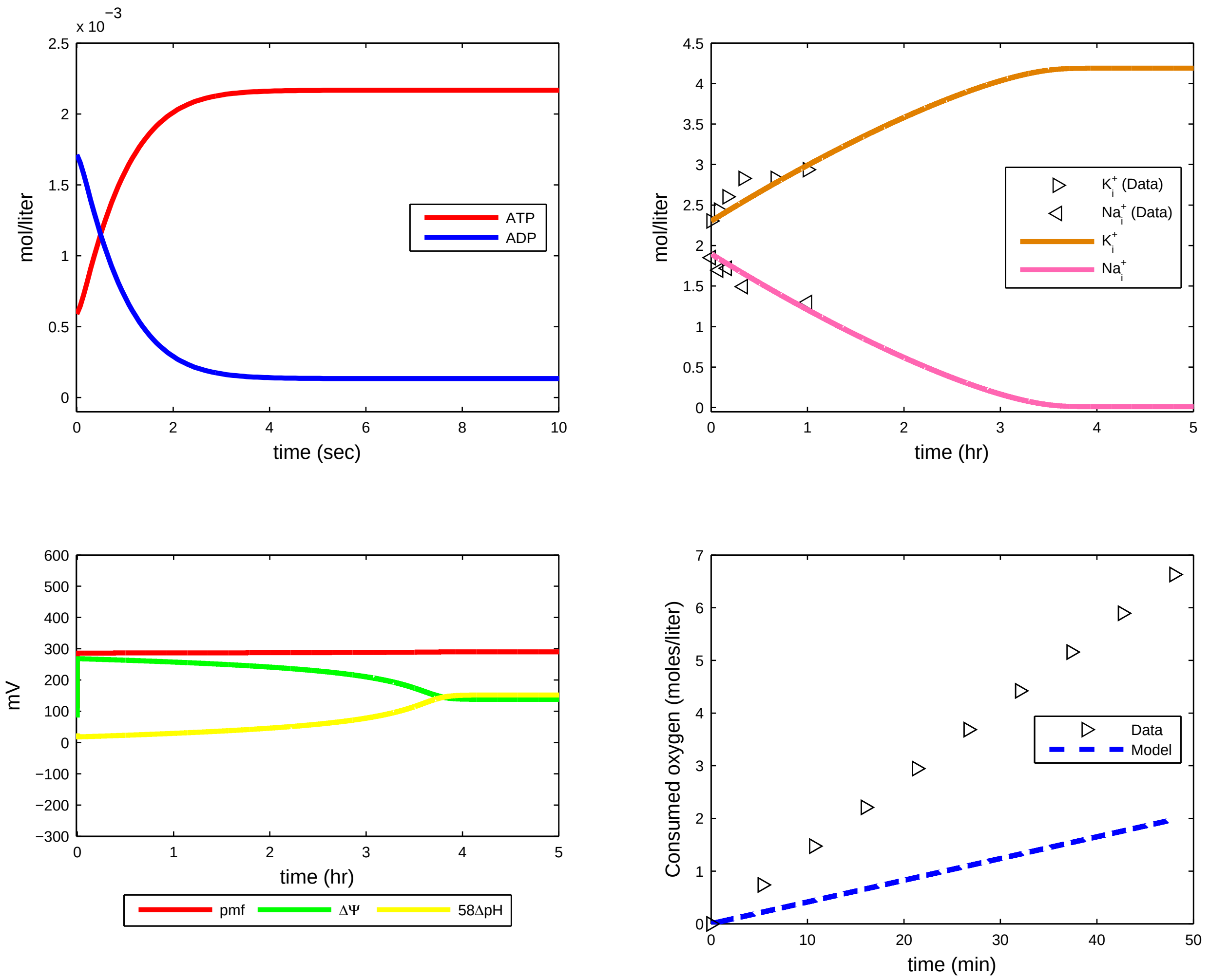

Fig D. Electroneutral model output using parameter set EN3 (Table 2).

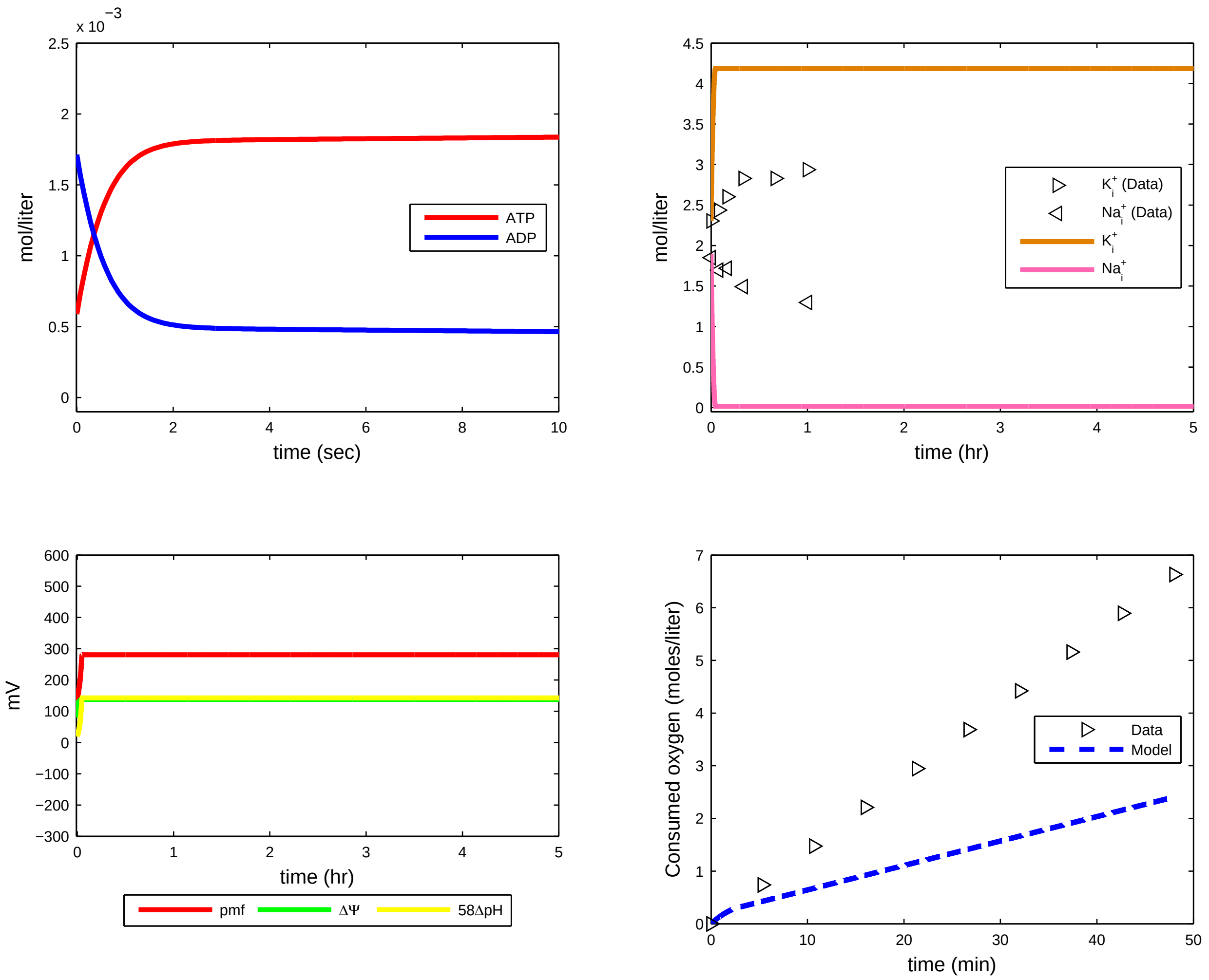

Fig E. Electroneutral model output using parameter set EN4 (Table 2).

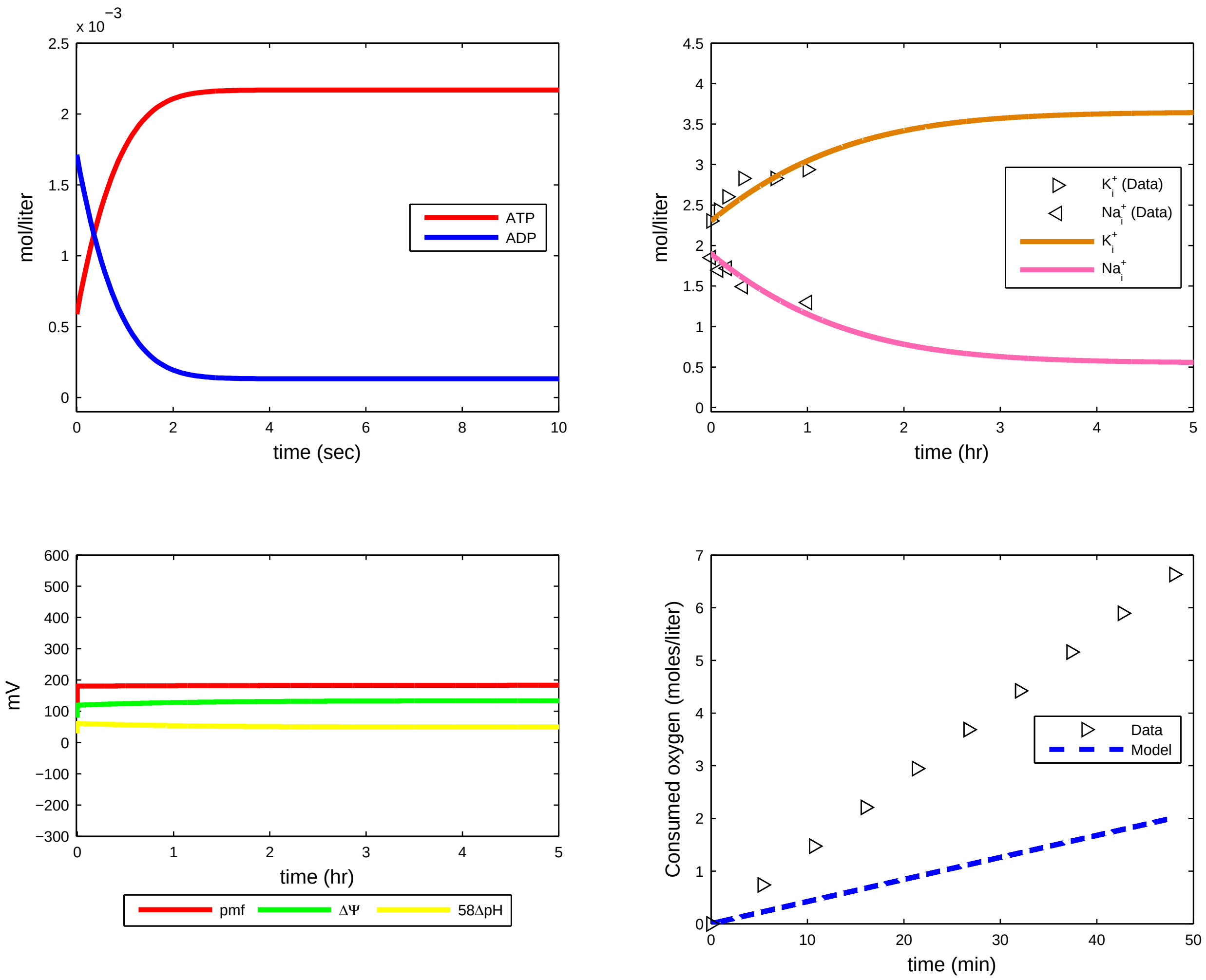

Fig F. Electroneutral model output using parameter set EN5 (Table 2).

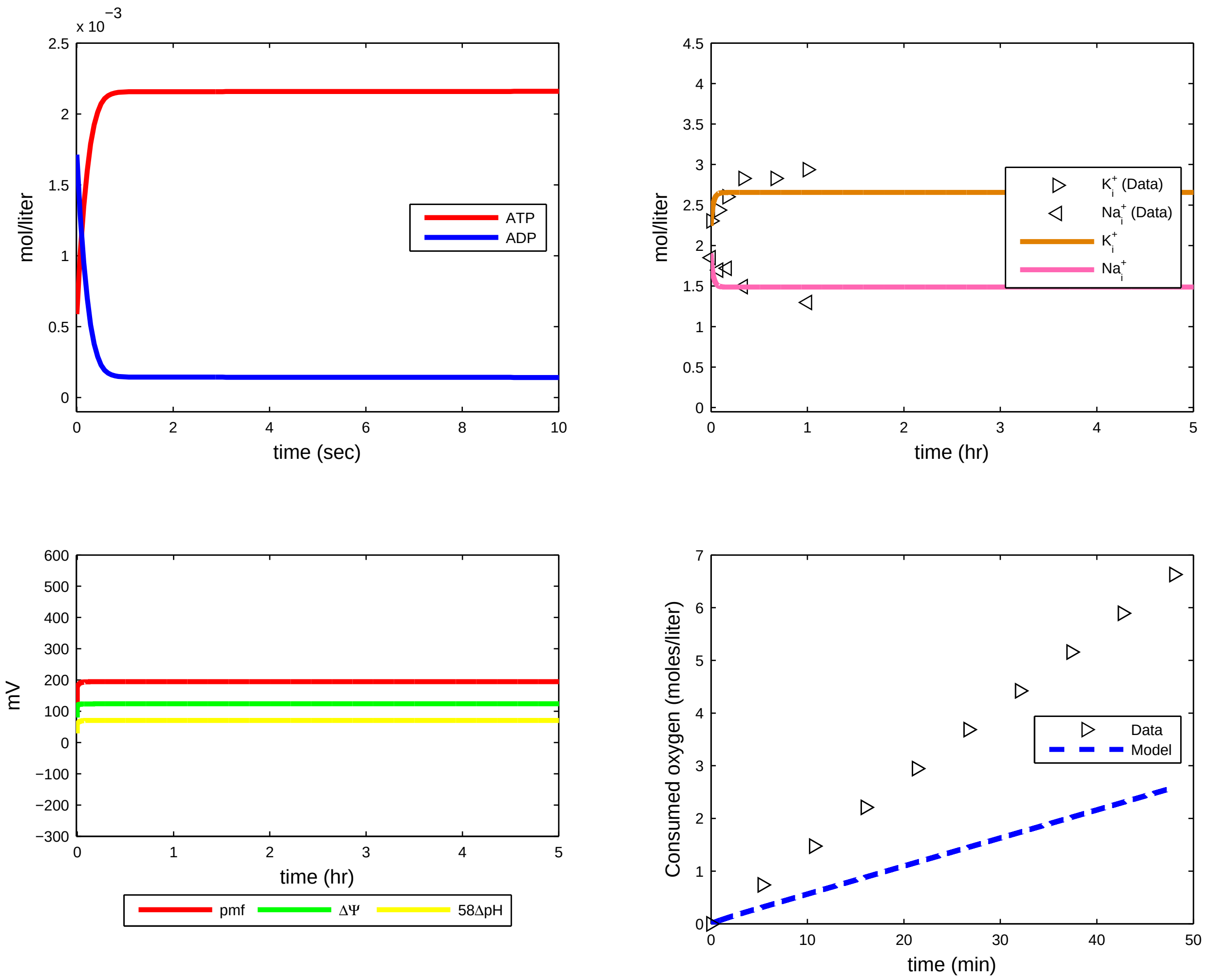

Fig G. Electrogenic model output using parameter set EG1 (Table 3).

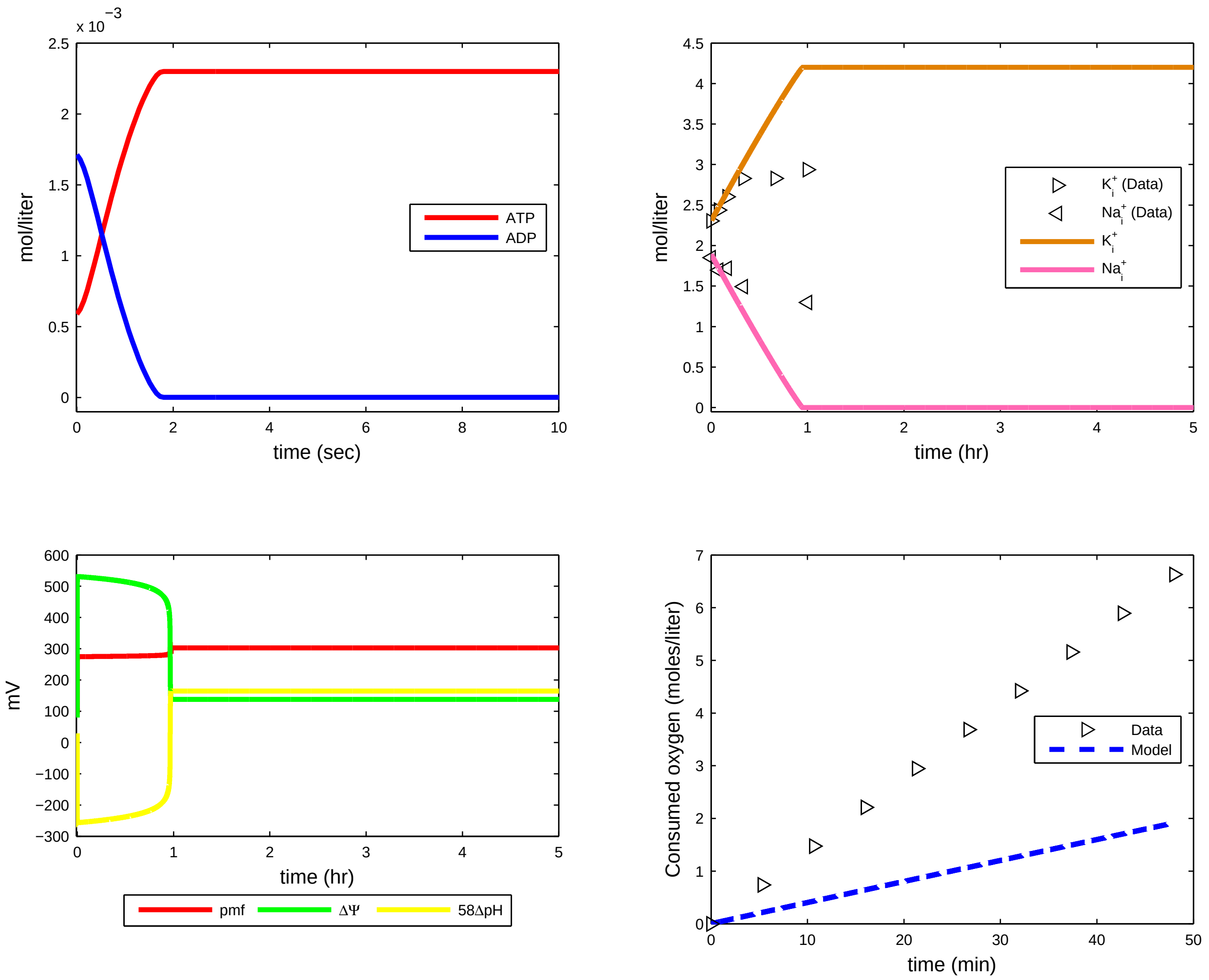

Fig H. Electrogenic model output using parameter set EG2 (Table 3).

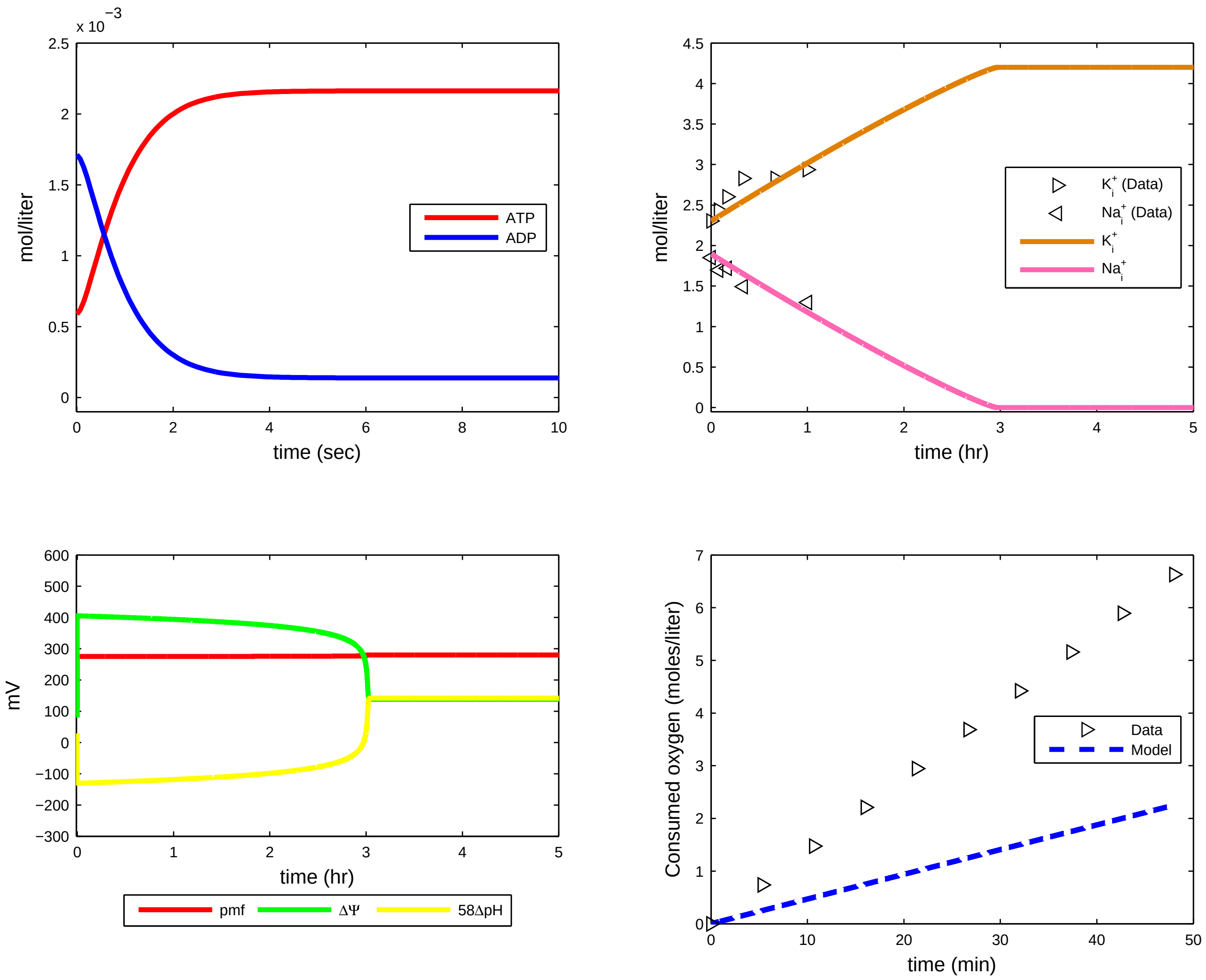

Fig I. Electrogenic model output using parameter set EG3 (Table 3).

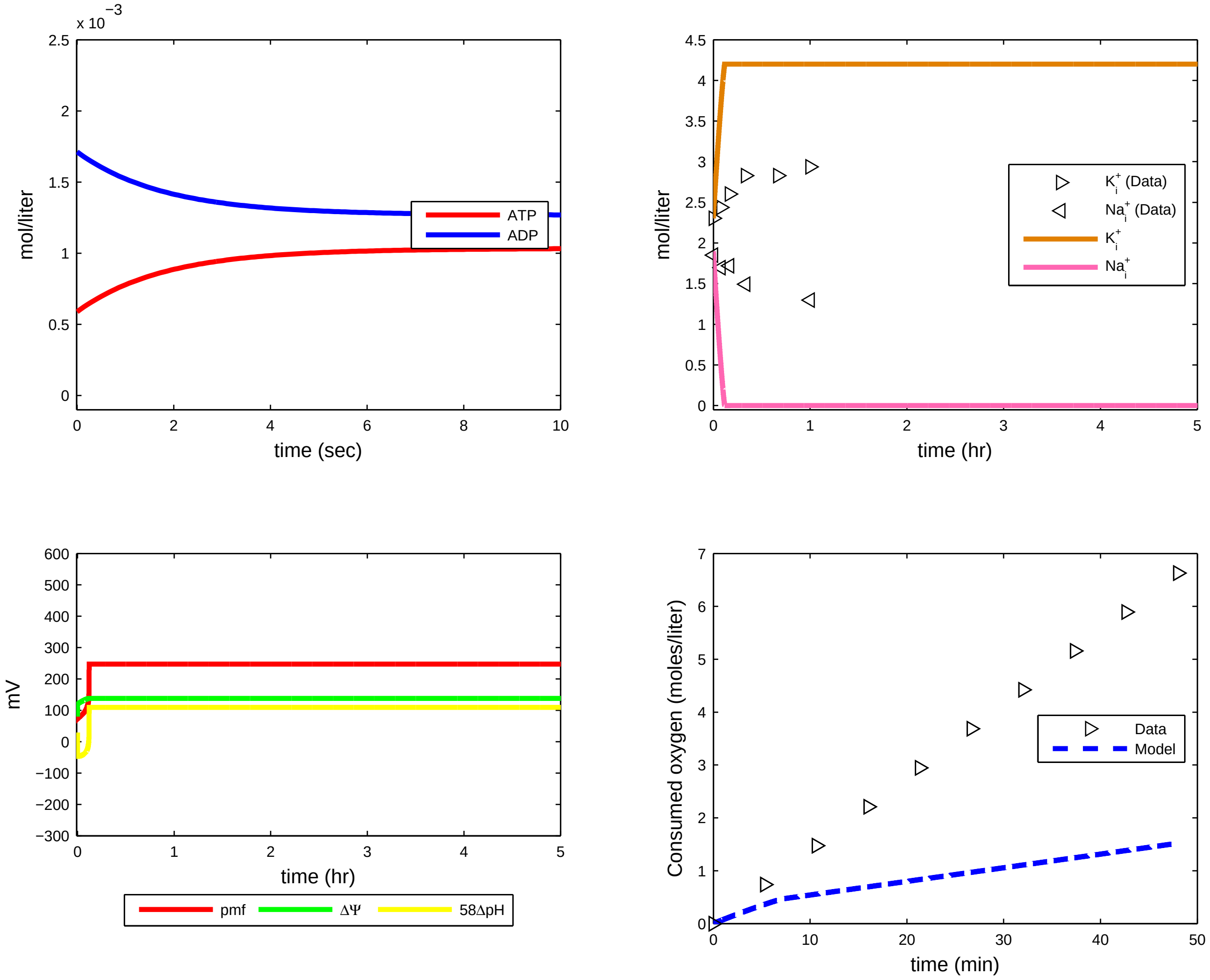

Fig J. Electrogenic model output using parameter set EG4 (Table 3).

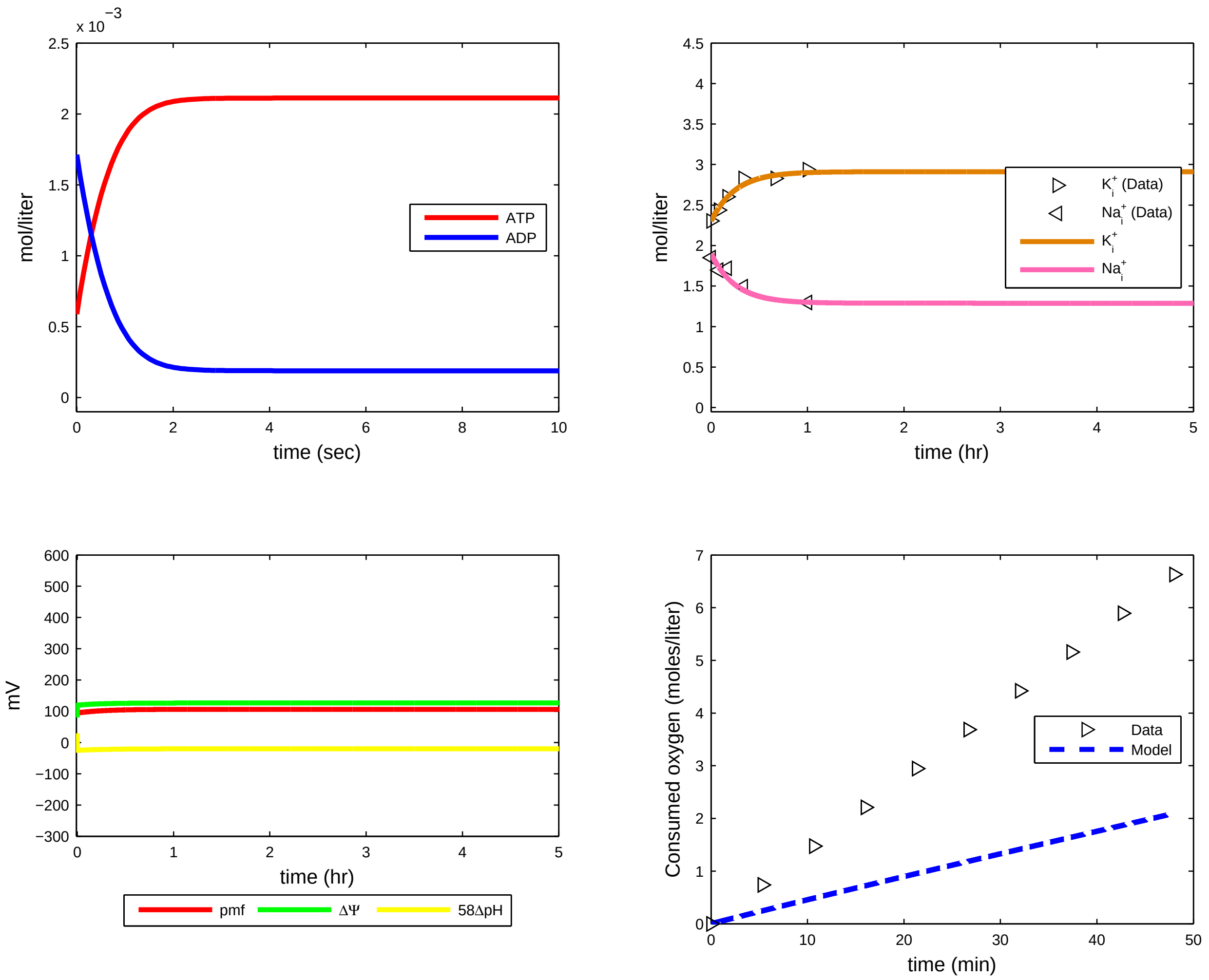

Fig K. Electrogenic model output using parameter set EG5 (Table 3).

References

1. Koch MK, Oesterhelt D. MpcT is the transducer for membrane potential changes in *Halobacterium salinarum*. Mol Microbiol. 2005;55(6):1681–1694.
2. Koch M. Investigations on halobacterial transducers with respect to membrane potential sensing and adaptive methylation. Munich, Germany: Ludwig-Maximilians-Universitaet Muenchen; 2005.
3. Michel H, Oesterhelt D. Light-induced changes in the pH gradient and membrane potential in *Halobacterium halobium*. FEBS Lett. 1976;65:175–178.
4. Hartmann R, Oesterhelt D. Bacteriorhodopsin-mediated photophosphorylation in *Halobacterium halobium*. Eur J Biochem. 1977;77:325–335.
5. Hartmann R, Sickinger HD, Oesterhelt D. Quantitative aspects of energy conversion in halobacteria. FEBS Lett. 1977;82:1–6.
6. Michel H, Oesterhelt D. Electrochemical proton gradient across the cell membrane of *Halobacterium halobium*: effect of N,N'-Dicyclohexylcarbodiimide, relation to intracellular

adenosine triphosphate, adenosine diphosphate, and phosphate concentration, and influence of the potassium gradient. Biochemistry. 1980;19(20):4607–4614.

7. Wagner G, Hartmann R, Oesterhelt D. Potassium uniport and ATP synthesis in *Halobacterium halobium*. Eur J Biochem. 1978;89:169–179.
8. Wende A, Furtwaängler K, Oesterhelt D. Phosphate-dependent behavior of the archaeon *Halobacterium salinarum* strain R1. J Bacteriol. 2009;191(12):3852–3860.
9. Engel MB, Catchpole HR. A microprobe analysis of inorganic elements in *Halobacterium salinarum*. Cell Biology International. 2005;29:616–622.
10. Oesterhelt D, Krippahl G. Light inihibition of respiration in *Halobacterium halobium*. FEBS Lett. 1973;32:72–76.
